# Overexpression of the Transcriptional Repressor Complex BCL-6/BCoR Leads to Nuclear Aggregates Distinct from Classical Aggresomes

**DOI:** 10.1371/journal.pone.0076845

**Published:** 2013-10-11

**Authors:** Elisabeth Buchberger, Miriam El Harchi, Dietmar Payrhuber, Anna Zommer, Dominic Schauer, Ingrid Simonitsch-Klupp, Martin Bilban, Christine Brostjan

**Affiliations:** 1 Department of Surgery, Medical University of Vienna, Vienna General Hospital, Vienna, Austria; 2 Clinical Institute of Pathology, Medical University of Vienna, Vienna General Hospital, Vienna, Austria; 3 Core Facility Genomics, Medical University of Vienna, Vienna General Hospital, Vienna, Austria; Boston University Medical School, United States of America

## Abstract

Nuclear inclusions of aggregated proteins have primarily been characterized for molecules with aberrant poly-glutamine repeats and for mutated or structurally altered proteins. They were termed “nuclear aggresomes” and misfolding was shown to promote association with molecular chaperones and proteasomes. Here, we report that two components of a transcriptional repressor complex (BCL-6 and BCoR) of wildtype amino acid sequence can independently or jointly induce the formation of nuclear aggregates when overexpressed. The observation that the majority of cells rapidly downregulate BCL-6/BCoR levels, supports the notion that expression of these proteins is under tight control. The inclusions occur when BCL-6/BCoR expression exceeds 150-fold of endogenous levels. They preferentially develop in the nucleus by a gradual increase in aggregate size to form large, spheroid structures which are not associated with heat shock proteins or marked by ubiquitin. In contrast, we find the close association of BCL-6/BCoR inclusions with PML bodies and a reduction in aggregation upon the concomitant overexpression of histone deacetylases or heat shock protein 70. In summary, our data offer a perspective on nuclear aggregates distinct from classical “nuclear aggresomes”: Large complexes of spheroid structure can evolve in the nucleus without being marked by the cellular machinery for protein refolding and degradation. However, nuclear proteostasis can be restored by balancing the levels of chaperones.

## Introduction

Deposition of protein aggregates indicates a failure in protein homeostasis (proteostasis) [1]. A system of interacting pathways which is termed “proteostasis network” functions to prevent or remove misfolded and aggregated proteins [2]. Molecular chaperones such as the heat shock proteins (HSPs) are central components of the proteostasis network, as they assist in protein folding and assembly; they also recognize incorrectly folded proteins and facilitate their degradation [3].

The initial protein structure is controlled co- and posttranslationally by interacting chaperones. Ribosome and nascent chain associated complexes prevent the newly synthesized peptides from non-native conformations by shielding hydrophobic amino acid residues [4,5]. While the classical HSP70 molecules do not bind directly to ribosomes, they may act on longer nascent peptide chains. Furthermore, they are the predominant cytosolic chaperones that facilitate protein folding posttranslationally [6]. Partially folded substrates and proteins which are inefficient targets of HSP70, are further transferred to other folding machineries: The chaperonins provide a cylindrical structure which facilitates protein folding by excluding cytosolic components [7,8]. The HSP90 system is particularly directed at the conformational control of signaling proteins [9]. Apart from the initial folding and assembly, many proteins require subsequent chaperone interactions to remain in a functionally active conformation. This so-called “conformational maintenance” is also promoted by the HSP70 family as shown in the bacterial setting [10].

If these structural guiding systems fail, the partially folded or misfolded proteins accumulate in amorphous aggregates, oligomers or amyloid-like fibrils [11]. To restore proteostasis the chaperones may then assist in the removal of aggregates by the ubiquitin-proteasome system (UPS) or by autophagy [12]. While the UPS is engaged by the cooperation of chaperones (e.g. HSP70 and HSP90) with ubiquitin ligases that recognize and label misfolded proteins by polyubiquitination [13], aggregated proteins which cannot be unfolded for proteasomal degradation may be eliminated by lysosome-based autophagy [14].

In addition to the cytosolic proteostasis network, components of subcellular compartments such as the endoplasmic reticulum and the Golgi provide a distinct local folding environment thereby supporting compartment-specific molecule conformations [15]. With respect to the nucleus, newly synthesized proteins larger than 40 kDa are actively imported via the nuclear pore complex [16]. Chaperones such as HSP70 and HSP90 can shuttle between the cytosol and nucleus [6,17]. Furthermore, the components of the UPS can be transported into the nucleus thereby providing the machinery for polyubiquitination and proteasomal degradation within this compartment [18].

The formation of particular structures termed “aggresomes” was originally detected in the cytosol and characterized as an aggregation process of misfolded proteins due to protein mutations, fusions, aberrant modifications or alterations by pH and reactive oxygen species [19]. Mechanistically, particles of misfolded proteins were described to be transported along microtubules (involving histone deacetylase 6, HDAC6) to microtubule-organizing centers where they coalesce to form large aggregates [20-22]. These so-called “aggresomes” are regarded as an intracellular storage form of accumulating proteins when the cellular degradation system is insufficient or overwhelmed [23]. In line with this notion, they are generally associated with molecular chaperones like heat shock proteins, ubiquitin and proteasomal subunits. The formation of these cellular inclusions is a hallmark of pathologies such as Alzheimer’s and Parkinson’s disease [24,25].

A subgroup of aggresomal diseases present with cytosolic as well as nuclear inclusion bodies. They mostly comprise neurodegenerative disorders and are collectively termed “polyQ diseases” [26]. Aggresome formation is induced by the aberrant amplification of CAG codons leading to extended poly-glutamine tracks in the disease-pertinent proteins [27,28]. The polyQ region generally seems to promote the tendency for protein self-aggregation. Similar to cytosolic aggresomes, nuclear inclusions are associated with molecular chaperones like HSP70, ubiquitin and proteasomal subunits [29,30].

Nuclear aggresomes were subsequently described for non-polyQ proteins and were mainly derived from artificial fusion constructs (GFP170*), from mutated or virally encoded proteins without extended glutamine stretches [31-33]. They were found to recruit chaperones (HSP70) and proteasomes, and to be associated with PML (promyelocytic leukemia) bodies [31,32]. The participation of PML bodies in proteasomal degradation was suggested to account for the close association of nuclear aggresomes with PML bodies. Of interest, protein deposition initiated in proximity to PML bodies, and fusion of smaller aggregates into larger structures was accompanied by spatial rearrangements of PML bodies [31]. The morphology of the nuclear inclusion varied with the investigated protein. The GFP170* fusion was described to generate dense spheroids with a complex internal structure including other nuclear components [31]. In contrast, missense mutations of the Epstein-Barr virus encoded ZEBRA (BamHI Z Epstein-Barr virus replication activator) homolog of AP-1 led to ring-like accumulations of mutant protein [32]. Cellular components like HSP70, PML and HDAC6 were entrapped at the inside, i.e. were surrounded by the aggresomal ring.

Mostly, nuclear aggresomes were based on proteins harboring non-endogenous (either mutated or pathogen-derived) sequences and were thus likely targets for the cellular machinery of protein sequestration, refolding and degradation. Aggregation was suggested to be triggered by the non-native protein structure and by misfolding. We will now present evidence for the formation of nuclear aggregates by overexpression of cellular proteins with unaltered (wildtype) amino acid sequence. Two components of a transcriptional repressor complex (BCL-6 and BCoR) can independently or jointly trigger the formation of ring-like nuclear inclusions which we have characterized with respect to aggregate formation, composition and cell response.

BCL-6 (B-cell lymphoma 6) was originally identified in translocations of non-Hodgkin’s lymphomas and was subsequently characterized as a potent transcriptional repressor with immunoregulatory function in germinal center development, memory T-cell generation and chemokine expression [34]. Its activity in endothelial cells (ECs) was reported to involve PPARδ (peroxisome proliferator-activated receptor delta) regulation in the context of inflammation [35]. BCL-6 is a 706 amino acid protein presenting with an N-terminal BTB/POZ domain for homo- and heterotypic protein interactions and a C-terminal zinc finger region for DNA binding [36]. It can directly recruit HDACs 4, 5 and 7 to exert its repressive effect on target gene expression [37]. In addition, BCL-6 associates with co-repressors like BCoR (BCL-6 interacting co-repressor) to enhance its regulatory properties [38]. BCoR is a large protein of 1721 amino acids with three ankyrin repeats but otherwise little homology to known protein sequences. It interacts with HDACs 1, 3, 4 and 5 [38] and engages in macromolecular complexes for epigenetic modifications to direct gene silencing [39]. BCoR has no DNA binding domain but is thought to function in association with transcription factors like BCL-6, AF9 or Sp1 [40,41]. Indeed, when overexpressed in primary microvascular endothelial cells, BCoR and BCL-6 co-localize in nuclear subdomains. Importantly, upon overexpression these proteins assemble in ring-like structures which are reminiscent of but partly distinct from nuclear aggresomes as characterized by the following analyses.

## Materials and Methods

### Ethics statement

This laboratory investigation involved primary cells retrieved from human tissue and was conducted according to the principles expressed in the Declaration of Helsinki. The isolation of cells from human tissue was approved by the institutional “Ethics Committee of the Medical University of Vienna” (#1123/2009); all volunteers or legal representatives gave written informed consent.

### Antibodies

Monoclonal anti-PML (sc-966), anti-Sp1 (sc-420), anti-PPARδ/β (sc-74440), anti-Hsp70 (sc-24), anti-ubiquitin (sc-8017) and polyclonal anti-BCL-6 (sc-858), anti-NFκB p65 (sc-109), anti-nucleolin/C23 (sc-13057) antibodies were purchased from Santa Cruz Biotechnology (Santa Cruz, CA). Anti-BCL-6 antibody (M7211) was obtained from Dako (Glostrup, Denmark), while anti-HA tag antibody (MMS-101P) was provided by Covance (Princeton, NJ). Anti-FLAG monoclonal antibody (637301) was derived from BioLegend (San Diego, CA). Anti-coilin (ab11822) and anti-nuclear pore complex proteins (ab24609) monoclonal antibodies, anti-BCoR (ab5276) and anti-lamin B1 (ab16048) polyclonal antibodies were obtained from Abcam (Cambridge, United Kingdom). Alexa Fluor 488 labeled donkey anti-rabbit, donkey anti-rat, donkey anti-goat IgG antibodies, Alexa Fluor 546 labeled goat anti-rabbit IgG antibody, Alexa Fluor 555 labeled donkey anti-mouse and donkey anti-goat IgG antibody, Alexa Fluor 633 labeled goat anti-rabbit and goat anti-mouse IgG antibodies were purchased from Invitrogen Corp. (Carlsbad, CA).

### Plasmids

The BCL-6 reporter plasmid as well as the cDNA expression constructs EFp-BCL-6 and EFp-BCoR-A based on the vector EFp-Link were generously provided by Micah Gearhart and Vivian Bardwell (University of Minnesota, MN) and have previously been described [38]. The CMV-Sp1 plasmid (12097; Robert Tjian; Howard Hughes Medical Institute, Berkeley, CA) as well as the expression constructs [42,43] for HDAC1-FLAG (13820), HDAC3-FLAG (13819), HDAC4-FLAG (13821), HDAC5-FLAG (13822), HDAC6-FLAG (13823), HDAC7-FLAG (13824), HSP70-1A (19456) and HSP90-HA (22487) were obtained from Addgene Inc. (Cambridge, MA). Expression construct pEGFP-BCL-6 [44] was a kind gift from Peter Jordan (Lisbon, Portugal). The pEGFP-C3 and pCMV-lacZ plasmids were purchased from Clontech (Mountain View, CA). Dr. W. C. Greene (Gladstone Institute, UCSF, CA) generously supplied pCMV4TΔp65. MeCP2-FLAG was kindly provided by Huda Zoghbi (Baylor College of Medicine, Houston, Texas).

### Cell culture and transfection

Primary ECs and fibroblasts as isolated to ≥ 98% purity from human foreskin by dispase digest were purified via anti-CD31 and anti-CD90 antibody coupled Dynabeads (Invitrogen), respectively. ECs were cultured in fibronectin-containing EGM2-MV growth medium (Lonza, Walkersville, MD) without VEGF supplementation in a 5% CO_2_ atmosphere. Fibroblasts were cultured in MEM medium supplemented with 20% fetal bovine serum (Linaris Corp., Wertheim, Germany), 1 mM sodium pyruvate, 2 mM L-glutamine, 100 U/ml penicillin and 100 µg/ml streptomycin (Invitrogen). All endothelial isolates were characterized by flow cytometry for EC characteristics, i.e., for CD31, CD34 expression and for E-selectin induction following stimulation with 100 ng/ml TNFα for 4 h. Fibroblast cultures were verified by the expression of CD90 and the absence of endothelial markers. The colon carcinoma cell line HT-29 (ATCC: HTB-38) was propagated in McCoy’s 5A medium (Invitrogen) supplemented with 10% fetal bovine serum, 2 mM L-glutamine, 100 U/ml penicillin and 100 µg/ml streptomycin. Cell transfection was generally carried out by electroporation: Cells were grown to 70-80% confluence, harvested and resuspended in RPMI1640 medium (PAA Corp., Pasching, Austria) containing 10% fetal bovine serum to obtain a cell count of 5x10^6^ per ml. A total of 20 µg plasmid DNA was added to 400 µl of cell suspension and cells were subsequently electroporated in a 4 mm cuvette at 200 V and 1200 µF with a Gene Pulser Xcell system (Bio-Rad Laboratories Inc., Hercules, CA). Cells were analyzed 4 to 24 h after transfection.

### Immunocytochemistry and confocal microscopy

Transfected cells were seeded on fibronectin-coated BioCoat Coverslips (BD-Biosciences, Franklin Lakes, NJ). After 4 to 24 h in culture cells were fixed in 4% paraformaldehyde for 10 min followed by permeabilization with 0.5% Triton X-100 for 7 min. The cells were washed extensively in phosphate-buffered saline (PBS) and blocked with 2% bovine serum albumin for 20 min. Primary antibody was added for 1 h. Cells were subsequently washed with PBS and incubated with Alexa Fluor conjugated secondary antibodies at a 1:1000 dilution of 2 mg/ml stocks. For nuclear staining 10 µg/ml Hoechst 33342 (Invitrogen) or 10 µM DRAQ5 (Biostatus Limited, Shepshed, United Kingdom) were added. Cells were washed again and fixed with 4% paraformaldehyde for 5 min. After a final washing step the cover slips were dried and mounted onto glass slides with ProLong^®^ Gold antifade reagent (Invitrogen). Stained samples were examined with a Zeiss LSM700 confocal microscope equipped with 40x/1.4 or 63x/1.4 Oil DIC objectives. Images were obtained and analyzed with Zen2009 software (Carl Zeiss AG, Oberkochen, Germany) and were further processed with Photoshop CS4 (Adobe Systems Inc., San Jose, CA). To quantitate BCL-6 expression levels the stained samples were scanned with a TissueFAXS 2.04 imaging system and analyzed with TissueQuest 4.01 software (TissueGnostics GmbH, Vienna, Austria).

### Cell cycle analysis by confocal microscopy

For CLSM-imaging transfected ECs were seeded on fibronectin-coated coverslips. After 18 h of cell culture BrdU was added to the medium for 1 hour. Cells were then fixed, permeabilized, blocked and treated with DNase followed by incubation with primary antibodies against BCoR or BCL-6. Secondary anti-goat or anti-rabbit Alexa Fluor 555 antibodies were used to detect BCoR or BCL-6 in combination with Alexa Fluor 488 labeled mouse anti-BrdU and Alexa Fluor 647 labeled rat anti-histone H3 (pS28) antibodies. Reagents were derived from the Cell Cycle Kit (BD Pharmingen^TM^, Franklin Lakes, NJ) and applied according to manufacturer’s instructions.

### Intracellular BCL-6/BCoR detection by flow cytometry

Transfected ECs were detached from the plate by trypsinization, fixed in 4% paraformaldehyde for 10 min, permeabilized with 0.5% Triton X-100 for 7 min and finally blocked with 20% FCS in PBS^-/-^. Cells were incubated with primary antibodies against BCL-6 or BCoR or the appropriate isotypic controls. Alexa Fluor 488 conjugated secondary antibodies were used for detection. The cells were finally resuspended in PBS^-/-^ containing 2.5% formaldehyde, 1% FCS and 0.5% NaN_3_ and were analyzed with a Gallios Flow cytometer (Beckman Coulter, Brea, CA).

### Analysis of EGFP fluorescence by flow cytometry

Endothelial cells transfected with the BCL-6-EGFP construct or the EGFP-C3 control vector were harvested 4 to 24 hours post transfection by trypsinization. Cells were resuspended in PBS^-/-^ containing 2.5% formaldehyde, 1% FCS and 0.5% NaN_3_ and were analyzed by flow cytometry for EGFP fluorescence.

### Detection of apoptotic cells by flow cytometry

Transfected ECs were seeded in 10 cm plates (1.5 x 10^6^ per plate). After 4 to 24 h, cells were harvested and fixed in 1 ml ice-cold ethanol (70%) for 30 min at 4°C. After centrifugation the cells were resuspended in cold PBS, and RNase A as well as propidium iodide was added to a final concentration of 50 µg/ml. Incubation for 15 min at 37°C was followed by analysis with the Gallios flow cytometer.

### Real-time RT-PCR

Total RNA was isolated from EC cultures with E.Z.N.A. MicroElute Total RNA Kit (Omega Bio-Tek Inc., Norcross, GA) according to the manufacturer’s instructions. 6 µl RNA were reverse transcribed with oligo(dT) primers using the QuantiTect Reverse Transcription Kit (Qiagen GmbH, Hilden, Germany) with a DNA elimination step prior to reverse transcription. The generated cDNA was diluted 1:25 before PCR analysis. Real-time PCR was performed with either MESA FAST qPCR MasterMix Plus for SYBR Assay Low ROX or qPCR MasterMix Plus Low ROX (Eurogentec, Searing, Belgium). The following primer sets were used for BCL-6 (300 nM forward primer 5’-CGGAGCCAGATTTGTACAGG-3’, 300 nM reverse primer 5’-CTGGCTTTTGTGACGGAAAT-3’), BCoR (450 nM forward primer 5’-AAGACTCCGAGATGTGCAAATTC-3’, 450 nM reverse primer 5’-TACTCGCATCTCTCACTTTCGTTC-3’, 200 nM 6-FAM/BHQ-1 labeled probe 5’-CAGCCGACTGGGAAAGGTTGAAAGG-3’) and housekeeping gene β-actin (450 nM forward primer 5’-CCTGGCACCCAGCACAAT-3’, 450 nM reverse primer 5’-GCCGATCCACACGGAGTACT-3’, 200 nM 6-FAM/BHQ-1 labeled probe 5’-ATCAAGATCATTGCTCCTCCTGAGCGC-3’). All primer sets spanned at least one exon/intron boundary. Each sample was assayed in duplicate with the GeneAmp 5700 Sequence Detection System (Applied Biosystems, Foster City, CA) for 45 cycles of 15 sec at 95 °C followed by 1 min at 60 °C. Denaturing curves were performed on all SYBR Green reactions to verify homogeneity of the amplified product. Transcript levels for BCL-6 and BCoR were calculated using the E^-ΔΔCt^ method. The efficiency of reactions was calculated according to the equation E = 10^-1^/slope [45]. Changes in mRNA expression upon EC transfection were calculated in relation to endogenous transcript levels (set to 1) of the mock-treated control.

For analysis of plasmid DNA in transfected ECs, a total amount of 6 ng extracted nucleic acid (including RNA and plasmid DNA) for SYBR Green reactions or 3.6 ng for TaqMan assays was directly subjected to real-time PCR amplification without reverse transcription of RNA. Absolute quantification was carried out using specific plasmids as standards.

### Reporter Gene Assay

ECs were transfected with 19 µg of the luciferase reporter construct (carrying 5 BCL-6 binding sites), 10 µg of EFp-BCL-6 expression plasmid or EFp-Link vector, and 1 µg of pCMV-lacZ control plasmid. Cell extracts were prepared 6, 12 and 24 hours after transfection and firefly luciferase as well as beta-galactosidase activity were determined by Dual Light Chemiluminescent Reporter Gene Assay (Applied Biosystems) in triplicate measurements with a Varioskan Flash Multimode Reader (Thermo Fisher Scientific Inc., Waltham, MA). Relative light units of luciferase activity were normalized to beta-galactosidase values and were expressed in relation to EFp-Link control samples set to 100%.

## Results

### Overexpression of BCL-6/BCoR leads to aggregate formation in the nucleus

**Figure 1:**
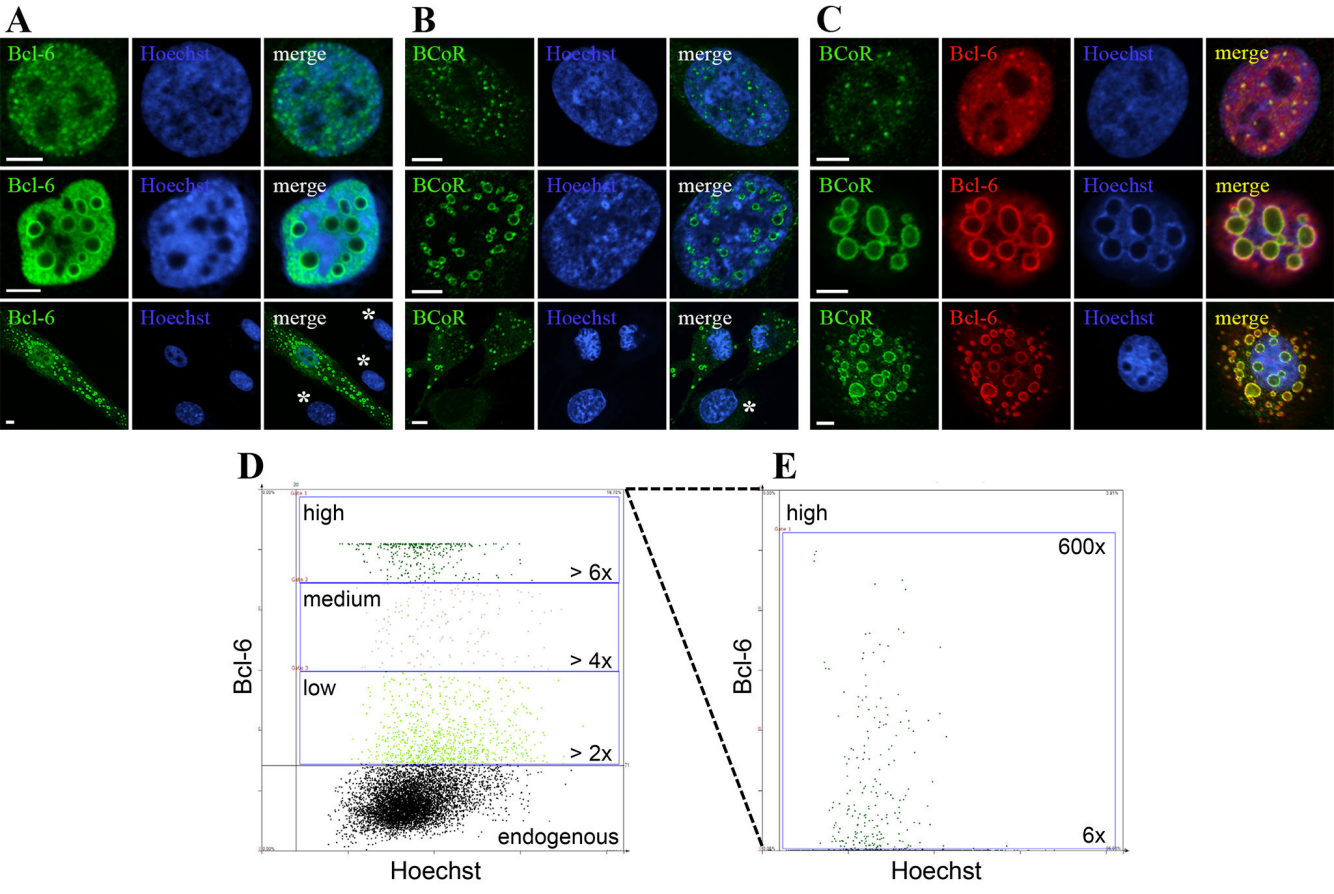
BCL-6/BCoR protein distribution upon overexpression in primary ECs. ECs were transfected with EFp-BCL-6 (A), EFp-BCoR-A (B) or a combination of the two expression plasmids (C). 24 hours after transfection cells were fixed and stained for CLSM imaging with antibodies against BCL-6 and/or BCoR. Hoechst 33342 was applied to counterstain nuclear DNA. Regions of co-localization of BCL-6 and BCoR protein are indicated in yellow; non-transfected cells are labeled by an asterisk. Scale bars: 5 µm. (D and E) ECs were transfected with 0, 2, 10 or 20 µg of EFp-BCL-6. The total amount of DNA was adjusted to 20 µg by the addition of EFp-Link control vector to standardize transfection conditions. Twelve hours later cells were subjected to immunostaining with BCL-6 antibody and Hoechst 33342 DNA stain and were analyzed with a TissueFAXS fluorescence detection system suited for the automated analysis of staining intensities in adherent cell cultures. The mock-treated control was used to determine the level of endogenous protein. BCL-6 expression in transfected cells is given in fold of mean control and divided in categories of low (> 2 fold), medium (> 4 fold) and high (> 6 fold) intensity. The scatter plot of DNA and BCL-6 fluorescence as recorded in 340 msec of detection is shown in (D) for the transfection with 10 µg EFp-BCL-6. For better resolution of high expressors, a second scan of 25 msec was performed (E). The frequency of transfected cells, the percentage of low, medium and high expressors and the occurrence of nuclear aggregates are listed in Table 1.

The transcriptional repressor complex BCL-6 and BCoR was investigated in primary ECs. Endogenous BCL-6 and BCoR proteins were hardly detectable by confocal microscopy, but proteins were readily revealed upon overexpression by transient cell transfection with cDNA expression constructs. We found BCL-6 as well as BCoR to be predominantly located in the cell nucleus accumulating in distinct patterns (Figure 1). BCL-6 staining was generally spread throughout the entire nucleus but condensed in small punctate structures in cells with moderate protein content (Figure 1A, top panel). ECs expressing high levels of BCL-6 formed large ring-like structures within the nucleus and infrequently showed additional aggregates in the cytosol (Figure 1A, middle and bottom panel). Overexpressed BCoR similarly developed into punctate structures or large rings within the nucleus and sporadically in the cytosol (Figure 1B). In contrast to BCL-6, BCoR protein showed little spreading throughout the nucleoplasm but was confined to aggregate structures. Co-expression of BCL-6 and BCoR resulted in co-localization in speckles (Figure 1C, top panel) and rings (Figure 1C, middle and bottom panel).

As the formation of large ring-like inclusions was a consequence of protein overexpression we further determined the prevalence of this phenotype in relation to the expression levels (Figure 1, D and E). When BCL-6 plasmid was titrated, the fraction of transfected endothelial cells remained constant (Table 1). However, the percentage of high expressors (> 6 fold of endogenous levels) increased proportionally to the amount of DNA applied. Cells with nuclear inclusions were only observed for high expressors: they reached BCL-6 levels elevated by more than 150 fold and a frequency of 50% at the highest DNA amount applied in all subsequent experiments.

**Table 1:** BCL-6 expression level and frequency of nuclear aggregates in relation to the amount of BCL-6 expression vector transfected into endothelial cells (compare Figure 1, D and E).

		**Low (> 2 fold)**		**Medium (> 4 fold)**		**High (> 6 fold)**	
	*% transfected*	% of transfected	% with aggregates	% of transfected	% with aggregates	% of transfected	% with aggregates
**20 µg** (n=6113)	*10.5*	37.1	0.0	14.3	0.0	48.6	54.1
**10 µg** (n=7897)	*15.2*	60.5	0.0	13.2	0.0	26.3	26.5
**2 µg** (n=10972)	*11.1*	70.3	0.0	13.5	0.0	16.2	13.2

ECs were transfected with 0, 2, 10 or 20 µg of EFp-BCL-6. The total amount of DNA was adjusted to 20 µg by the addition of EFp-Link control vector to standardize transfection conditions. Twelve hours later cells were subjected to immunostaining with BCL-6 antibody and Hoechst 33342 DNA stain and were analyzed with a TissueFAXS fluorescence detection system suited for the automated analysis of staining intensities in adherent cell cultures. The mock-treated control was used to determine the level of endogenous protein. BCL-6 expression in transfected cells is given in fold of mean control and divided in categories of low (> 2 fold), medium (> 4 fold) and high (> 6 fold) intensity. The overall frequency of transfected cells (% transfected) as well as the percentage of low, medium and high expressors within the transfected population (% of transfected) are listed. Furthermore, the populations of low, medium and high expressors were manually screened for the occurrence of nuclear aggregates (% with aggregates).

To address the question whether protein accumulation in large ring-like aggregates was specific for BCL-6/BCoR or a general phenomenon upon overexpression of transcription factors, ECs were transfected with plasmids for the transcriptional activators NF-κB, Sp1 or the repressor MeCP2. While all regulators localized to the nucleus and were partly enriched in nuclear subdomains, no formation of large aggregates was observed (Figure S1). Apart from the apparent protein specificity, we found BCL-6/BCoR aggregation to occur irrespective of the transfected cell type and the immunocytochemical detection method applied (Figures S2 and S3).

### BCL-6/BCoR aggregates form large, hollow spheres

**Figure 2:**
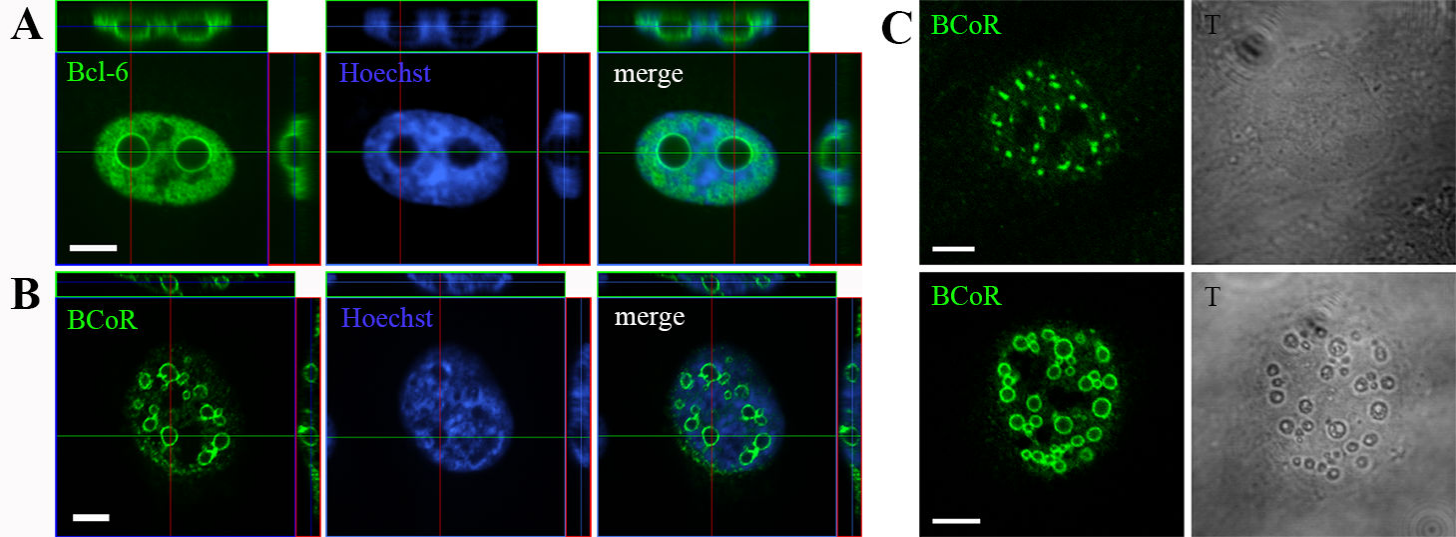
Structure and nuclear reorganization of BCL-6/BCoR inclusions. ECs were transfected with EFp-BCL-6 (A) or EFp-BCoR-A (B and C) expression plasmid and processed with Hoechst 33342 DNA stain and antibodies against BCL-6 or BCoR for CLSM imaging after 24 hours. (A and B) Compilation of Z-stack series into orthogonal projections. (C) Comparison between fluorescence and light transmission (gray scale) images of BCoR-A transfected cells. Scale bars: 5 µm.

When investigating the ring-like BCL-6/BCoR structures by 3-dimensional Z-stack the aggregates were found to be spherical and largely excluding nuclear components like DNA from the inside (Figure 2, A and B). Occasionally, weak staining for BCL-6 or BCoR was detected within spheres. Light transmission images confirmed that the formation of large aggregates led to remarkable structural changes of the nucleus (Figure 2C) which were observed for BCL-6 and/or BCoR overexpression.

As the exclusion of DNA from large nuclear structures was reminiscent of nucleoli we tested whether BCL-6/BCoR aggregates were indeed perinucleolar. Co-staining for C23 (nucleolin) revealed that ring-like BCL-6/BCoR structures did not associate with or substantially alter the nucleoli detected in transfected cells (Figure S4).

### BCL-6/BCoR aggregates exhibit a time-dependent evolvement from small to large structures

**Figure 3:**
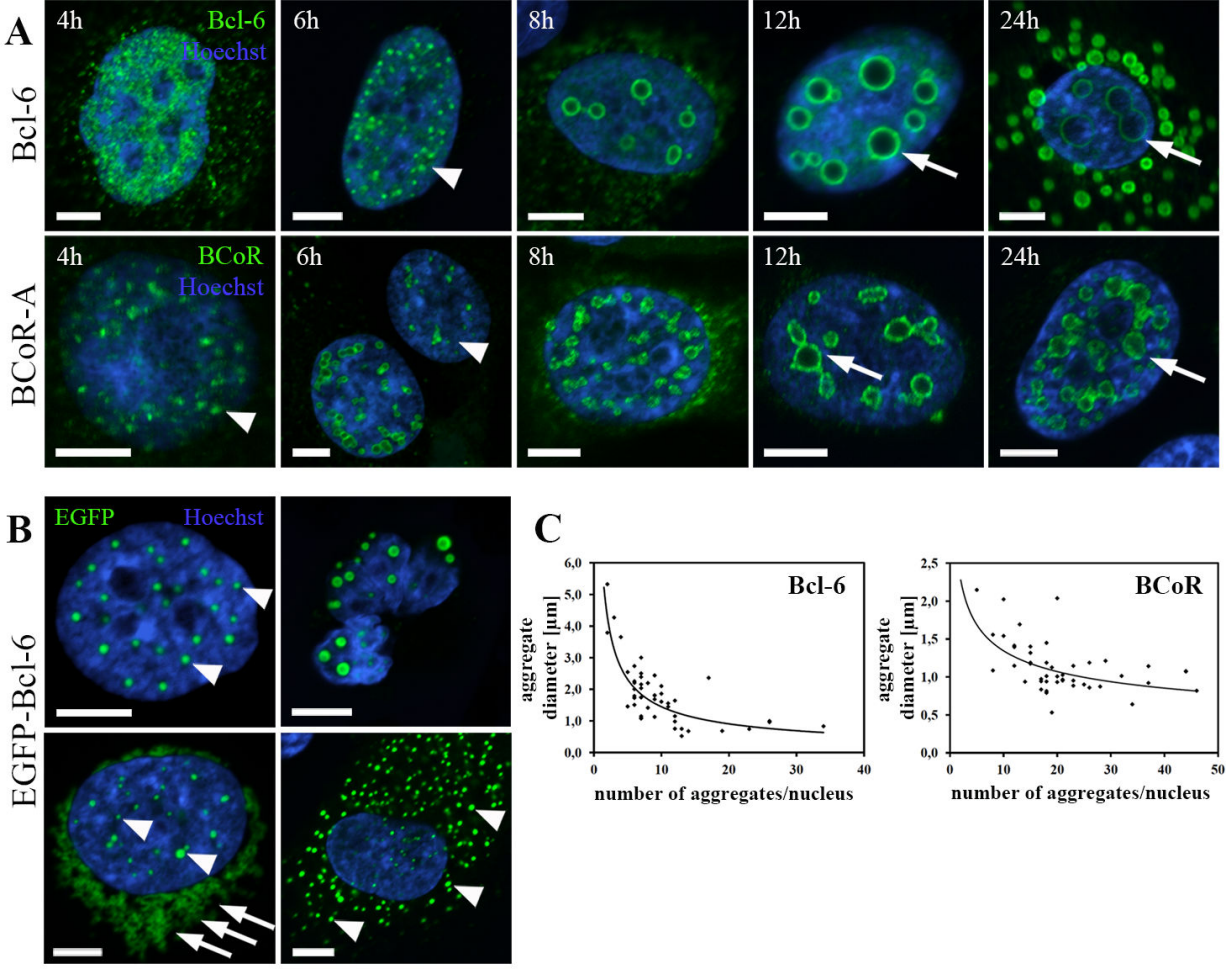
Time course of aggregate formation. (A) ECs were transfected with EFp-BCL-6 or EFp-BCoR-A plasmid and processed for CLSM imaging after 4 to 24 hours using antibodies against BCL-6 and BCoR, respectively. Hoechst 33342 was applied to counterstain nuclear DNA. Arrowheads indicate small, punctate structures; arrows point to large spheres of aggregates. (B) EC transfection with EGFP-BCL-6 expression plasmid was followed by cell processing for CLSM imaging at 8 to 12 hours after transfection. Arrowheads indicate small, punctate structures in nucleus and cytosol; arrows point to diffuse perinuclear accumulations. The upper right image illustrates a large spherical EGFP-BCL-6 aggregate in a cell with fragmented nucleus. (C) Diameter and number of aggregates per nucleus were determined at 12 h after EC transfection with BCL-6 or BCoR-A expression plasmid. A statistically significant, negative correlation according to Spearman test was established for BCL-6 (rho = -0.697, p < 0.01) and BCoR (rho = -0.503, p < 0.01). Scale bars: 5 µm.

When monitoring BCL-6/BCoR protein expression over a time frame of 4 to 24 hours (Figure 3A), we found that small, punctate accumulations were predominantly observed at 4 to 6 hours after transfection. Nuclear, ring-like structures with increasing diameter were detected from 8 to 24 h indicating a time-dependent formation of large inclusions. Furthermore, cytosolic BCL-6/BCoR “rings” were exclusively found at late time points.

Nuclear BCL-6 spheres ranged between 0.5 to 5.3 µm with an average diameter of 1.8 µm. While smaller spheres (< 1 µm) were found in nuclei containing more than 10 aggregates, large spheres (> 3 µm) occurred only in numbers of 1 to 5 per nucleus. The inverse correlation between number and diameter of nuclear aggregates supports the notion that large spheroids are formed by coalescence of smaller aggregates (Figure 3C). BCoR spheres were smaller than BCL-6 spheres with an average diameter of 1.1 µm ranging between 0.5 and 2.1 µm. Comparably, an inverse relation between number and diameter of nuclear BCoR aggregates was observed.

To possibly substantiate the coalescence of aggregate structures by live cell imaging, we tested an EGFP fusion variant of BCL-6 for nuclear aggregation. Of note, EGFP-tagged BCL-6 greatly differed from the wild type protein as the overexpression resulted predominantly in small punctate aggregates and diffuse perinuclear accumulations (Figure 3B). The formation of large spheroid inclusions was greatly reduced and only detected for cells with deformed and fragmented nuclei indicating cytotoxicity. To quantitatively compare aggregate formation between wild type proteins and the EGFP-fusion variant, the frequency of staining patterns at 12 h post transfection was determined (Table 2). While 34% of BCL-6 and 52% of BCoR expressing cells showed large ring-like structures in apparently intact nuclei, only 13% of EGFP-BCL-6 positive cells embodied large spheroid aggregates with predominantly fragmented nuclei (12%). Large cytosolic spheroids were entirely absent from cells expressing EGFP-BCL-6 in contrast to wildtype BCL-6 (13%) and BCoR (3%).

**Table 2:** Protein expression pattern at 12 hours after EC transfection with expression plasmids for EGFP-BCL-6, BCL-6 or BCoR.

	**Large Ring-like Structures**			**Small Punctate Structures**			**Diffuse**
	nuclear	nuclear (fragmented nuclei)	nuclear + cytosolic	nuclear	nuclear + cytosolic	nuclear + perinuclear	nuclear
EGFP-BCL-6(n=120)	2/120 (1.7%)	14/120 (11.7%)	-	30/120 (25.0%)	31/120 (25.8%)	18/120 (15.0%)	25/120 (20.8%)
**BCL-6** (n=102)	22/102 (21.6%)	-	13/102 (12.7%)	20/102 (19.6%)	-	-	47/102 (46.1%)
**BCoR-A** (n=69)	34/69 (49.3%)	-	2/69 (2.9%)	33/69 (47.8%)	-	-	-

### BCL-6/BCoR aggregates do not inhibit cell cycle progression, but spheroid structures are not observed during mitosis

**Figure 4:**
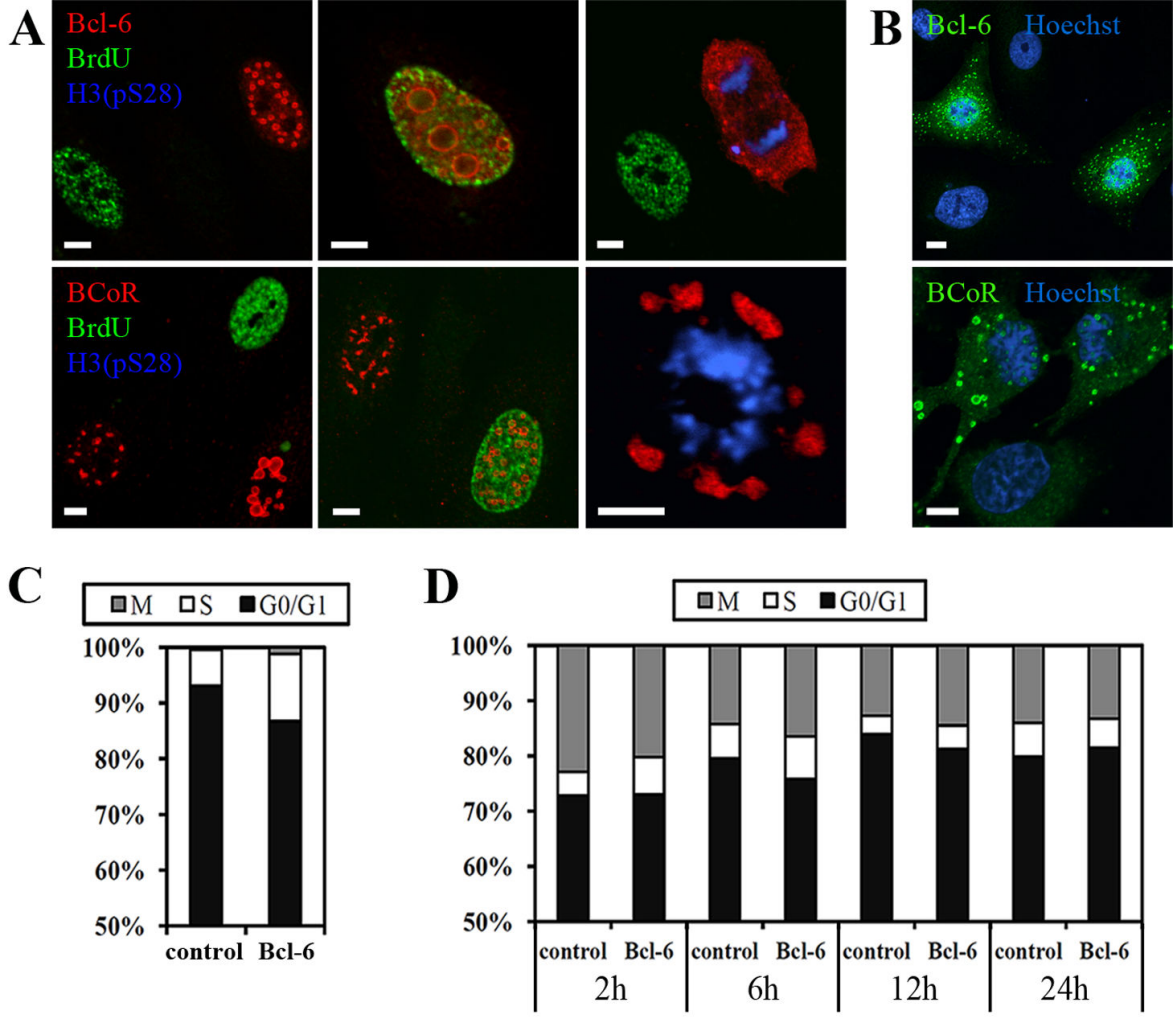
BCL-6/BCoR aggregates during cell cycle progression. (A) Confocal images of ECs transfected with EFp-BCL-6 or EFp-BCoR-A, cultured for 18 h and subsequently loaded with BrdU for 1 h. Cells were immunostained with antibodies against BCL-6 or BCoR, and with antibodies against BrdU and H3(pS28) to detect ECs in S-phase or mitosis, respectively. Cells in G0/G1 phase are reflected by the absence of BrdU and H3(pS28) staining. (B) Confocal images of EFp-BCL-6 or EFp-BCoR-A transfected ECs, processed with antibodies against BCL-6 or BCoR and with DNA stain Hoechst 33342 to illustrate the appearance of cytosolic aggregates in neighboring cells. (C) Based on confocal images exemplified in (A), 2000 cells were counted and classified in G0/G1, S or M/G2 phase. The BCL-6 positive and negative (non-transfected) cell populations were compared for their cell cycle distribution. (D) Cell cycle distribution as established by PI stain and flow cytometry of ECs at 2 to 24 h after transfection with EGFP-BCL-6 or EGFP-C3 control plasmid. Only EGFP positive cells were evaluated. Scale bars: 5 µm.

When staining for cells in G0/G1, S-phase or mitosis, we found that BCL‑6/BCoR overexpression was detectable in all cell cycle phases (Figure 4A). While large spheroid inclusions were evident in cells of G0/1 or S-phase, ring-like structures were not observed in mitotic cells indicating that they may be reorganized during cell division. Of interest, the concomitant appearance of nuclear and cytosolic “rings” was generally detected in adjoining cells of the G0/G1 phase (Figure 4B). This observation supports the notion that BCL-6/BCoR protein is “released” during the mitotic breakdown of the nuclear envelope and may reassemble in spheroid structures of the post-mitotic nucleus or cytosol.

To verify that aggregate formation does not alter cell cycle distribution, cells of G0/G1, S-phase or mitosis were quantitatively assessed in CLSM (confocal laser scanning microscopy) images at 24 h post-transfection (Figure 4C). We found no blockade of cell cycle progression in the BCL-6 positive as compared to the negative (non-transfected) population. Similar results were obtained when evaluating the cell cycle distribution by flow cytometry at 2 to 24 h after EC transfection with the EGFP-BCL-6 fusion construct versus EGFP control (Figure 4D).

### BCL-6/BCoR aggregates are not associated with heat shock proteins or marked by ubiquitin

As previously reported, nuclear as well as cytosolic aggresomes are commonly associated with heat shock proteins and are marked by ubiquitination for proteasomal degradation. Since these features are considered hallmarks of aggresome formation, we investigated HSP70, HSP27 and ubiquitin distribution in ECs overexpressing the transcriptional repressors BCL-6/BCoR. Unexpectedly, we found no association of BCL‑6 or BCoR aggregates with heat shock proteins and ubiquitin.

**Figure 5:**
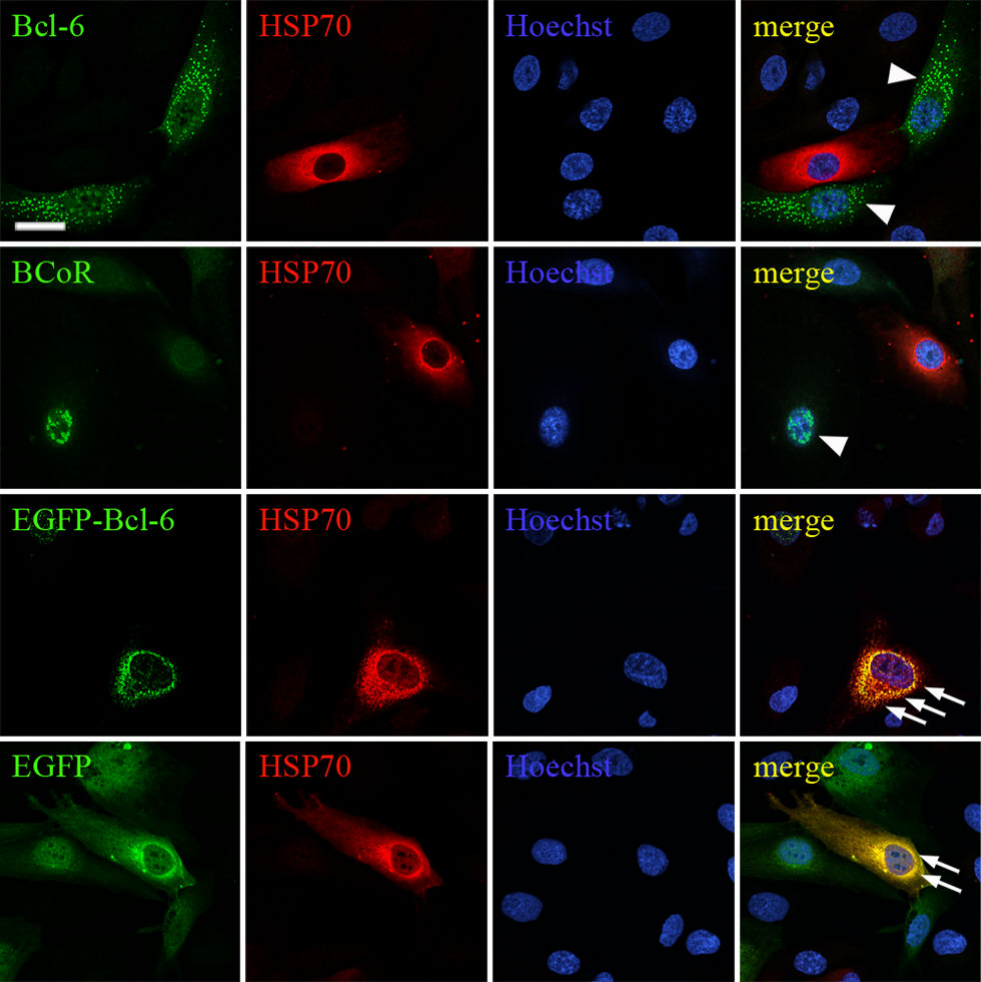
Endogenous HSP70 expression in cells with BCL-6/BCoR aggregates. ECs were transfected with EFp-BCL‑6, EFp-BCoR-A, EGFP-BCL-6 or EGFP-C3 plasmid and processed for CLSM imaging 12 hours after transfection. All cells were stained with α-HSP70 antibody and Hoechst 33342. EFp-BCL-6 and EFp-BCoR-A transfected cells were further immunostained with antibodies against BCL-6 or BCoR. BCL‑6/BCoR aggregates without HSP70 association are indicated by arrow heads. Regions of co-localization are indicated by arrows and marked in yellow. Scale bar: 10 µm (applying to all panels).

In non-transfected ECs, HSP70 levels were generally low with a few cells showing strong HSP70 expression in the cytosol. This pattern was not altered by BCL‑6/BCoR overexpression, i.e. HSP70 was not induced and did not co-localize with BCL-6/BCoR aggregates (Figure 5). Of interest, the EGFP-BCL-6 fusion protein triggered a strong induction and co-localization of HSP70 in the nucleus and cytosol. Notably, this effect was also observed for the majority of ECs transfected with EGFP control plasmid.

With respect to HSP27, ECs showed constitutively high levels in the cytosol which remained unaffected by BCL-6/BCoR inclusions (Figure S5). The fusion of EGFP to BCL-6 triggered a weak accumulation and association of HSP27 with cytosolic EGFP-BCL-6 aggregates.

Similarly, staining of transfected ECs for ubiquitin (Figure S6) showed no co-localization with BCL-6/BCoR aggregates, whereas the perinuclear accumulations of EGFP-BCL-6 protein seemed to be associated with ubiquitin.

### Overexpression of HSP70 reduces the formation of large BCL-6/BCoR aggregates which is partly reversed by proteasome blockade

**Figure 6:**
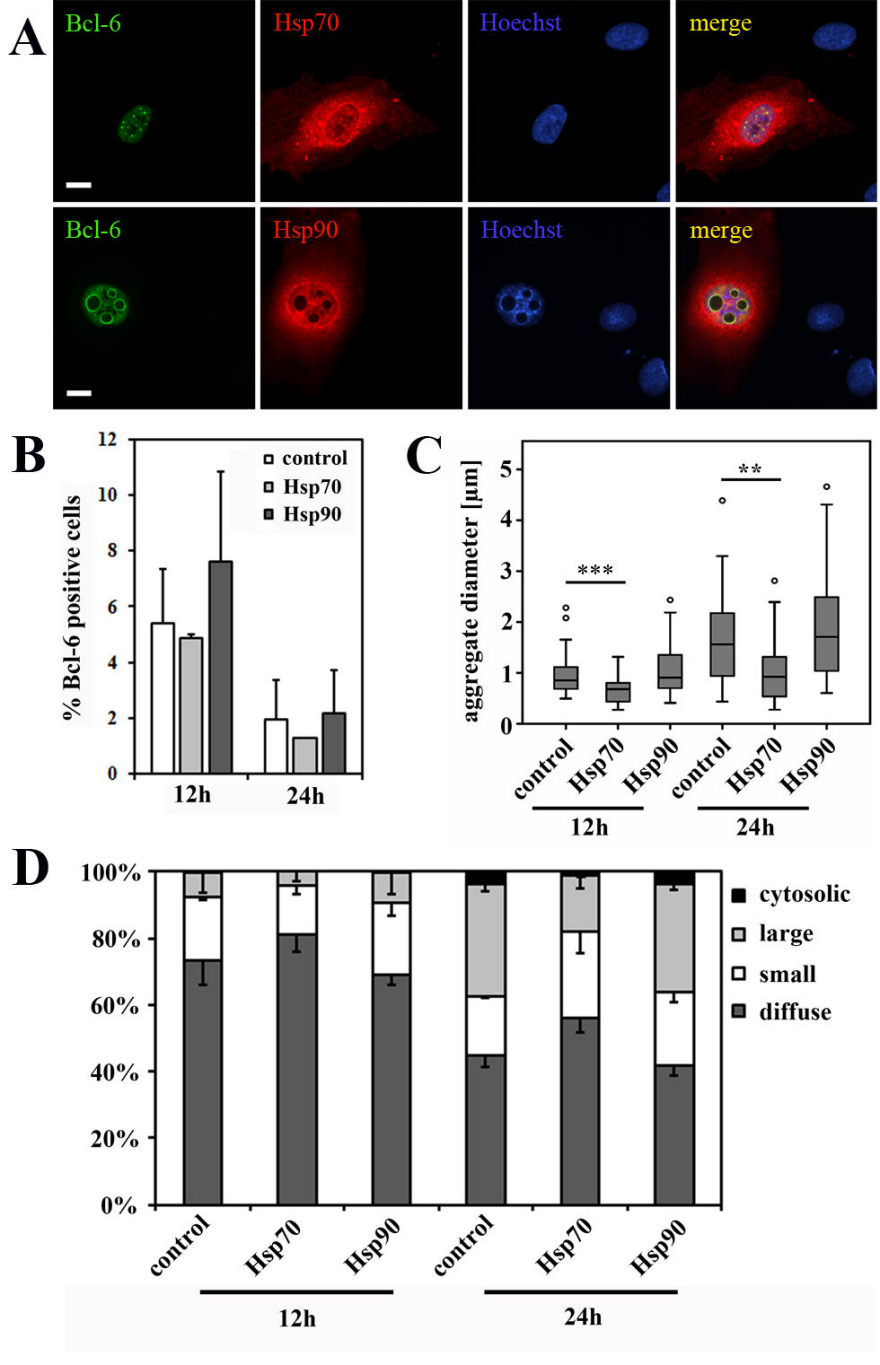
BCL-6 aggregate formation upon co-expression of exogenous HSP70 and HSP90. ECs were transfected with EFp-BCL‑6 in combination with HSP70-1A or HSP90-HA expression plasmid or with EFp-Link control vector. After 12 and 24 hours cells were stained for CLSM imaging with antibodies against BCL-6 and HSP70 or with antibodies against BCL-6 and HA-tag for HSP90 detection. Hoechst 33342 was applied to counterstain nuclear DNA. (A) CLSM images demonstrate the concomitant overexpression of BCL-6 with HSP70 or HSP90 at 24 hours after transfection (scale bars: 10 µM). (B) The percentage of BCL-6 positive cells was determined from four randomly acquired tile scans (40x objective) covering approximately 700 cells. Mean values and standard deviations from two independent experiments are given; T-test revealed no statistically significant differences. (C) 35 randomly selected nuclei with BCL-6 aggregations were analyzed (per condition) for the diameter of their aggregates. The boxplot illustrates the distribution of acquired values with a statistically significant reduction in aggregate diameter upon co-expression of HSP70 after 12 h (T-test; p < 0.001) and 24 h (T-test; p = 0.001). (D) 300 BCL-6 positive cells (per condition) were classified according to their nuclear BCL-6 expression pattern into diffuse (no aggregation), small punctate, large spherical and additional cytosolic aggregates. Mean values and standard deviations were calculated from 2 independent experiments. The frequency of large, spherical aggregates was significantly reduced upon co-expression of BCL-6 and HSP70 after 24 h (T-test; p = 0.031). *, p < 0.05; **, p < 0.01; ***, p < 0.001.

**Figure 7:**
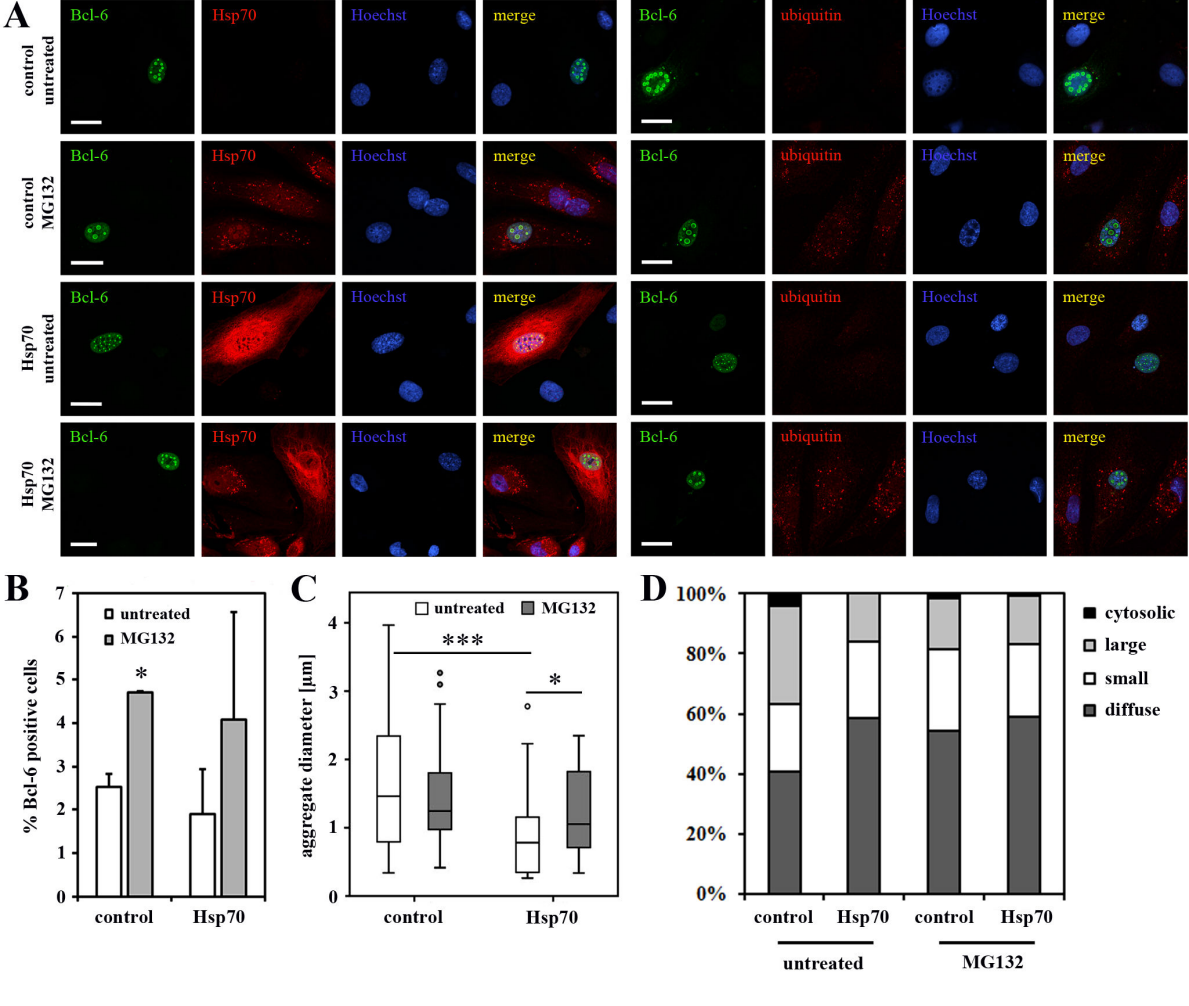
The effect of proteasome blockade on BCL-6 aggregate formation. ECs were transfected with EFp-BCL‑6 in combination with HSP70-1A or EFp-Link control vector. Three hours after transfection the cells were exposed to 0.5 µM MG132 or left untreated. At 24 h after transfection ECs were stained for CLSM imaging with antibodies against BCL-6 and HSP70 or with antibodies against BCL-6 and ubiquitin, including Hoechst 33342 as DNA counterstain. (A) Microscopy images illustrate the expression pattern of BCL-6, HSP70 and ubiquitin (scale bars: 20 µM). (B) The percentage of BCL-6 positive cells was determined from four randomly acquired tile scans (40x objective) covering approximately 700 cells. Mean values and standard deviations from two independent experiments are given. The frequency of BCL-6 positive cells was found to be significantly increased upon addition of MG132 (T-test; p = 0.01). (C) 35 randomly selected nuclei with BCL-6 aggregations were analyzed (per condition) for the diameter of their aggregates. The boxplot illustrates the distribution of acquired values with a statistically significant reduction in aggregate diameter upon co-expression of HSP70 (T-test; p < 0.001) which was partly reversed by MG132 treatment (T-test; p = 0.028). (D) 300 BCL-6 positive cells (per condition) were classified according to their nuclear BCL-6 expression pattern into diffuse (no aggregation), small punctate, large spherical and additional cytosolic aggregates. Mean values and standard deviations were calculated from 2 independent experiments. The frequency of large, spherical aggregates was significantly reduced upon co-expression of BCL-6 and HSP70 (T-test; p = 0.025). The effect of MG132 had no statistical significance. *, p < 0.05; **, p < 0.01; ***, p < 0.001.

Based on the finding that BCL-6/BCoR inclusions were apparently not marked by the cellular machinery for protein refolding and degradation, we evaluated whether the enhanced expression of chaperones might restore proteostasis. We co-transfected ECs with expression plasmids for BCL-6 and HSP70-1A or HSP90, two prominent members of the HSP family known to enter the nucleus. Both chaperones were detected at high levels within the nucleus. While overexpression of HSP90 did not affect the accumulation of BCL-6 aggregates, the elevated levels of HSP70 significantly reduced the frequency of large nuclear inclusions and the median diameter in aggregate size (Figure 6). When proteasomal degradation was inhibited by the addition of MG132, the effect was partly reversed (Figure 7): aggregate size rather than frequency was significantly higher. MG132 treatment per se resulted in the accumulation of ubiquitinated proteins within cells and enhanced the expression of endogenous HSP70 which was, however, not sufficient to reduce BCL‑6 aggregate formation under proteasome blockade. Conversely, MG132 treatment increased the number of cells with detectable BCL-6 expression but did not further enhance the frequency or diameter of nuclear aggregates formed upon BCL-6 overexpression.

### BCL-6/BCoR aggregates are affected by the co-expression of HDACs

**Figure 8:**
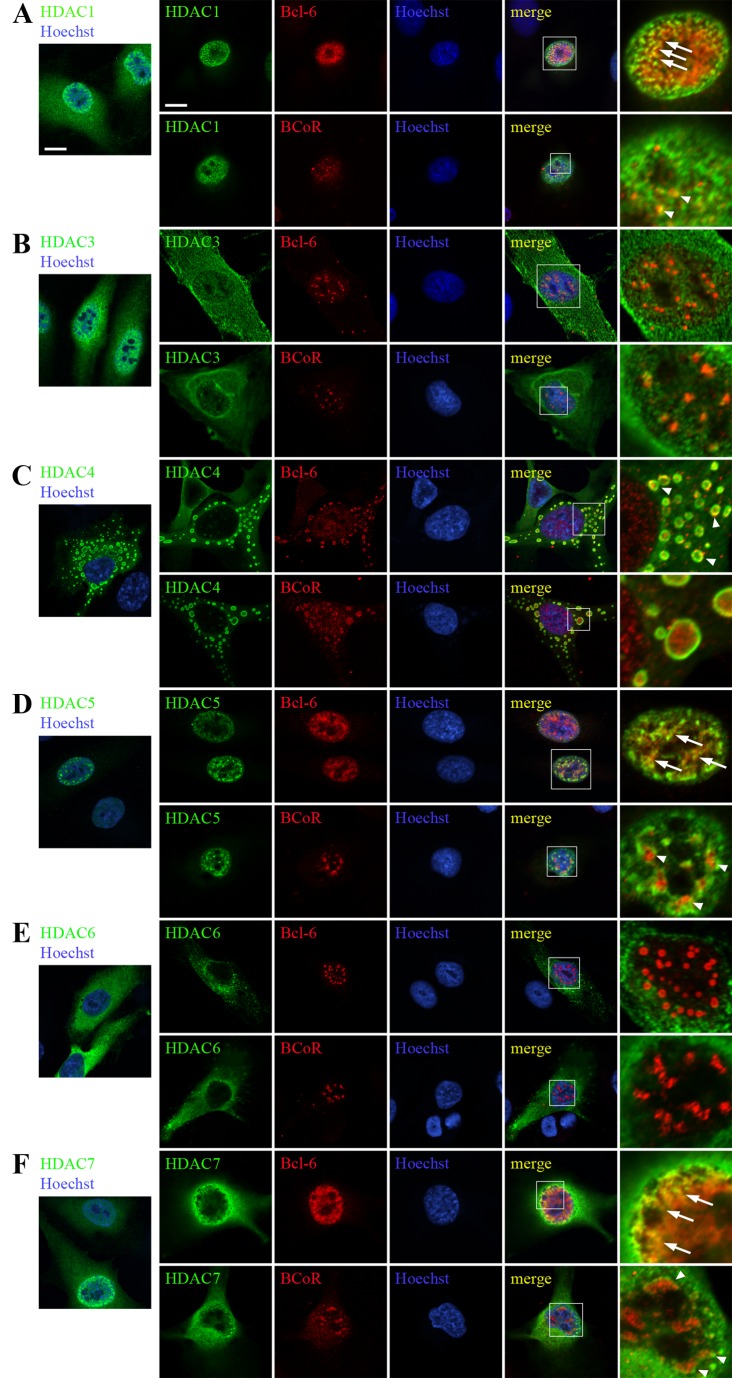
Association of overexpressed BCL-6/BCoR protein with HDACs. ECs were transfected with FLAG-tagged expression constructs of HDAC1 (A), HDAC3 (B), HDAC4 (C), HDAC5 (D), HDAC6 (E) or HDAC7 (F) and processed for CLSM imaging 12 hours after transfection with an antibody directed against the FLAG-tag and with DNA stain (Hoechst 33342). The first lane illustrates HDAC distribution without concomitant BCL-6/BCoR overexpression. All other images were derived from ECs exposed to co-transfection of HDAC expression constructs and EFp-BCL-6 or EFp-BCoR-A plasmid. Cells were additionally stained with antibodies against BCL‑6 or BCoR. The last panel shows magnifications of indicated regions (white rectangles). Arrows and arrowheads in the merged pictures point to regions of co-localization of HDACs with BCL-6 or BCoR. Scale bars: 5 µm (applying to all panels).

Since transcriptional repression by BCL-6/BCoR crucially involves histone deacetylation and previous co-immunoprecipitation experiments have documented the interaction between BCL-6/BCoR and HDACs [37,38], we investigated the association of BCL‑6/BCoR inclusions with distinct HDAC members. ECs were transfected with FLAG-tagged expression constructs for HDAC1, HDAC3, HDAC4, HDAC5, HDAC6 or HDAC7 with or without the addition of BCL-6 and BCoR-A expression plasmid (Figure 8). Twelve hours after transfection strong expression of FLAG-tagged HDAC1, HDAC3 and HDAC7 was detectable in the nucleus along with lower levels in the cytosol. HDAC5 was exclusively nuclear, whereas HDAC4 and HDAC6 were predominantly found in the cytoplasm. Of note, HDAC4 formed large, ring-like structures within the cytosol.

When BCL-6 or BCoR were co-expressed with HDACs 1, 4, 5 or 7, the occurrence of nuclear BCL-6/BCoR inclusions was largely inhibited indicating that the association of BCL-6/BCoR with HDACs may interfere with aggregate formation. Conversely, larger (ring-like or punctate) aggregations of BCL-6 or BCoR were only found in combination with HDAC3 and HDAC6 which had no evident co-localization/association with BCL-6/BCoR proteins (Figure 8B and 8E).

BCoR generally showed a more punctate expression pattern than BCL-6 in the presence of HDACs. HDACs 1, 5 and 7 were located in the vicinity of nuclear BCoR speckles or were overlapping with nuclear BCL-6 protein (Figure 8A, 8D and 8F). In contrast, both BCL-6 and BCoR were redistributed to the cytosol by concomitant HDAC4 expression. While BCL-6 was located to the rim of cytosolic HDAC4 “rings”, BCoR seemed to fill the inside. Thus, overexpression of distinct HDACs substantially affected BCL-6/BCoR protein distribution and aggregate formation.

Two other reported interaction partners of BCL-6 and BCoR were investigated with respect to BCL-6/BCoR inclusions. The endogenous transcription factors PPARδ and Sp1 were located in small nuclear speckles, but did not co-localize with BCL‑6/BCoR aggregates (Figure S7).

The apparent association of Bcl-6/BCoR aggregates with histone deacetylases led us to further test their functionality in transcriptional repression. ECs were co-transfected with a luciferase reporter construct carrying five BCL-6 binding sites and with the EFp-BCL-6 expression plasmid or EFp-Link control vector (Figure S8). Repression of luciferase reporter gene activity by 70-80% was observed at 12 and 24 h post transfection, at time points of BCL-6 aggregation. While these results argue for transcriptional functionality despite BCL-6/BCoR aggregation, they do not exclude the possibility that transcriptional inhibition is exerted by non-aggregated protein at the time points investigated.

### BCL-6/BCoR aggregates associate with nuclear bodies and result in their spatial rearrangement

**Figure 9:**
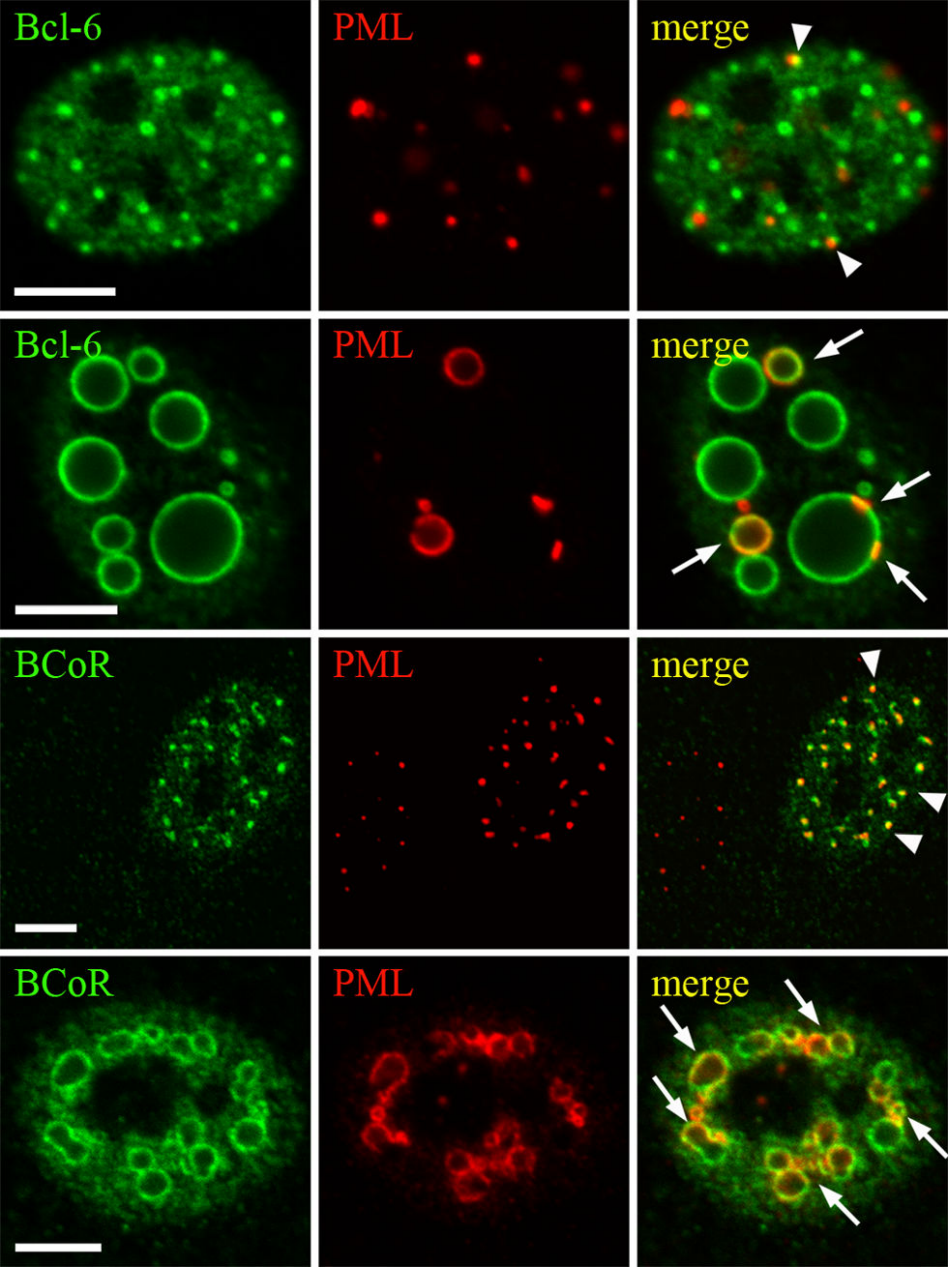
Impact of BCL-6/BCoR aggregates on the nuclear distribution of PML bodies. ECs were transfected with EFp-BCL-6 or EFp-BCoR-A DNA and processed for CLSM imaging after 24 hours. Antibodies against BCL-6 or BCoR were combined with α-PML antibody for immunostaining. Association of PML bodies with small punctate BCL-6/BCoR aggregates is marked by arrowheads. Arrows indicate incorporation of PML protein in the rim of large spheroid inclusions. Scale bars: 5 µm.

We further addressed the relation of BCL-6/BCoR inclusions with nuclear bodies involved in transcriptional regulation such as PML and Cajal bodies. PML bodies were well defined nuclear structures appearing in 10 to 30 foci per nucleus in non-transfected ECs. Upon overexpression, small as well as large BCL-6/BCoR inclusions were found to associate with nuclear PML bodies (Figure 9). While small punctate BCL-6/BCoR aggregates showed partial overlap with PML bodies, larger aggregates led to a remarkable redistribution of PML bodies into the ring-like structures. Notably, not all BCL-6/BCoR inclusions were accompanied by PML bodies and PML protein was generally restricted to the outside of spheroids.

The nuclear distribution of Cajal bodies was also altered by BCL‑6/BCoR overexpression (Figure S9) i.e. the detected coilin component was induced to accumulate in BCL-6/BCoR aggregates.

### BCL-6/BCoR aggregates are generally not associated with components of the nuclear envelope but seem to acquire coverage upon emergence in the cytosol

Based on the observation that BCL-6/BCoR inclusions led to a remarkable reorganization of the nuclear structure, the impact on the nuclear “boundaries” seemed of further interest. The nuclear envelope consists of an outer and an inner nuclear membrane containing the nuclear pore complex of nucleoporins (NUPs), and it is tightly connected to the underlying layer of nuclear lamins [46]. We therefore performed co-stainings of BCL-6/BCoR with antibodies against NUPs and lamin B1.

**Figure 10:**
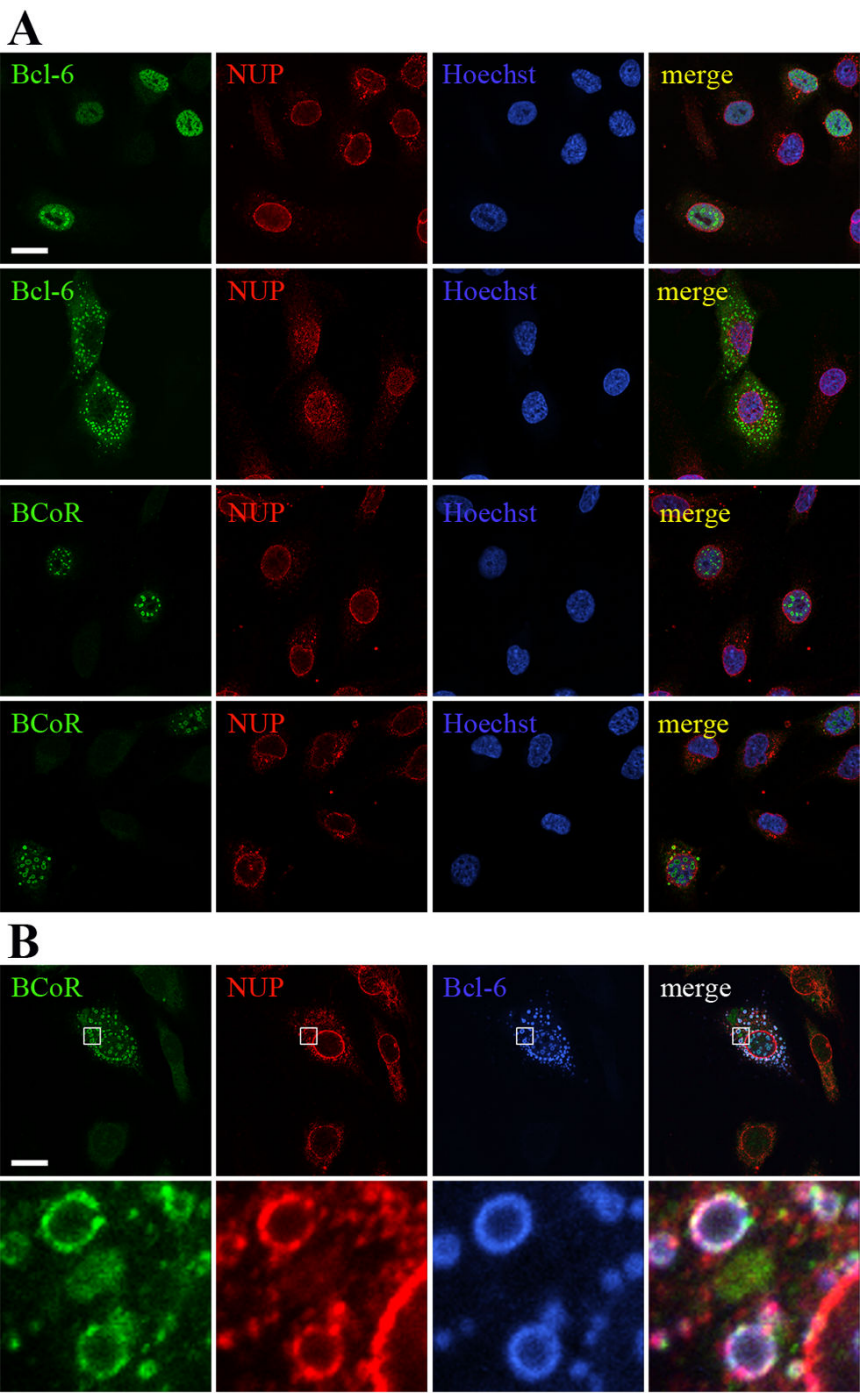
Relation of BCL-6/BCoR aggregates to components of the nuclear pore complex. ECs were transfected with EFpBCL-6 or EFp-BCoR-A (A) or with a combination of both plasmids (B). Cells were processed after 12 hours with Hoechst 33342 DNA stain and with antibodies against NUPs, BCL-6 and BCoR for CLSM imaging. Scale bars: 5 µm (applying to all panels). The last lane shows magnifications of the indicated regions (white rectangles).

NUPs were generally not associated with nuclear BCL-6 or BCoR aggregates (Figure 10A). Interestingly, we found co-localization of NUPs with cytosolic inclusions which was specifically observed when both transcription factors were co-expressed (Figure 10B). NUP association with cytosolic BCL‑6/BCoR spheroids may thus be based on NUP shuttling between nucleus and cytoplasm or might reflect aggregate “release” from the nucleus to the cytosol.

Furthermore, we found lamin B1 to be differentially affected by BCL-6 and BCoR inclusions (Figure S10). Lamins are known as a platform for sequestering transcription factors at the nuclear periphery by direct protein interactions [47]. Lamin B1 seemed to be simply displaced by BCL-6 spheroids whereas apparent accumulation and co-localization of lamin B1 was recorded for BCoR aggregates. This indicates a selective interaction of lamin B1 with BCoR but does not argue for lamin B1 involvement in aggregate formation.

### BCL-6/BCoR expression is tightly controlled at the transcript and protein level

**Figure 11:**
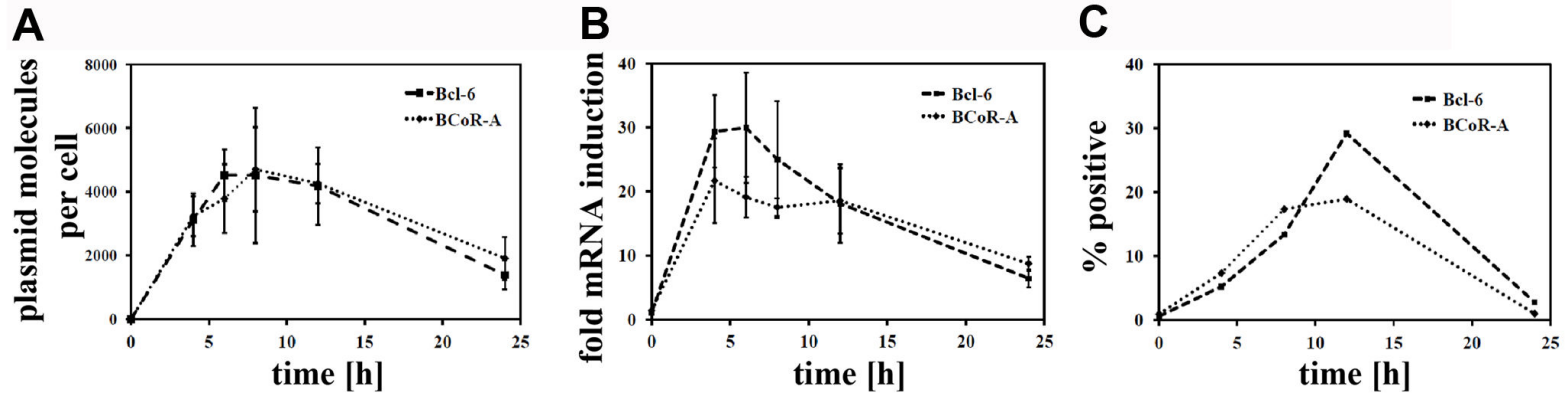
Comparison of plasmid DNA, mRNA and protein content of BCL‑6/BCoR transfected ECs. Cells were transfected with EFp-BCL-6 or EFp-BCoR-A expression plasmid. (A) The amount of plasmid per cell was determined by qPCR of cell extracts. The results represent mean values and standard deviations of 3 independent experiments. (B) The increase in mRNA in relation to endogenous levels was measured by qRT-PCR of the corresponding RNA extracts. (C) Protein expression was evaluated by flow cytometry of permeabilized cells and is given as the percentage of BCL-6/BCoR positive ECs.

Since the extent of expression was generally low for exogenous BCL-6/BCoR when compared to EC transfection with control molecules like EGFP, we aimed to further investigate the regulatory mechanisms of BCL-6/BCoR expression. Thus, transfected cells were analyzed for plasmid DNA content, mRNA and protein levels. We found BCL-6 and BCoR expression plasmids to be highly abundant in ECs (Figure 11A). Peak levels of 2000 to 6000 molecules were detected per cell at 8 h post transfection. The DNA concentration decreased to half within 24 h. In comparison, the amount of transcribed mRNA was only moderately increased by 20 to 40 fold over endogenous BCL-6/BCoR mRNA levels (Figure 11B). The mRNA peak at 4 to 6 h was followed by a decline to nearly baseline within 24 h after transfection. With respect to protein expression, we evaluated the percentage of BCL-6/BCoR positive cells by flow cytometry. Peak levels of 20-30% were generally reached between 8-12 h with a marked disappearance of BCL-6/BCoR positive cells at 24 h (Figure 11C).

**Figure 12:**
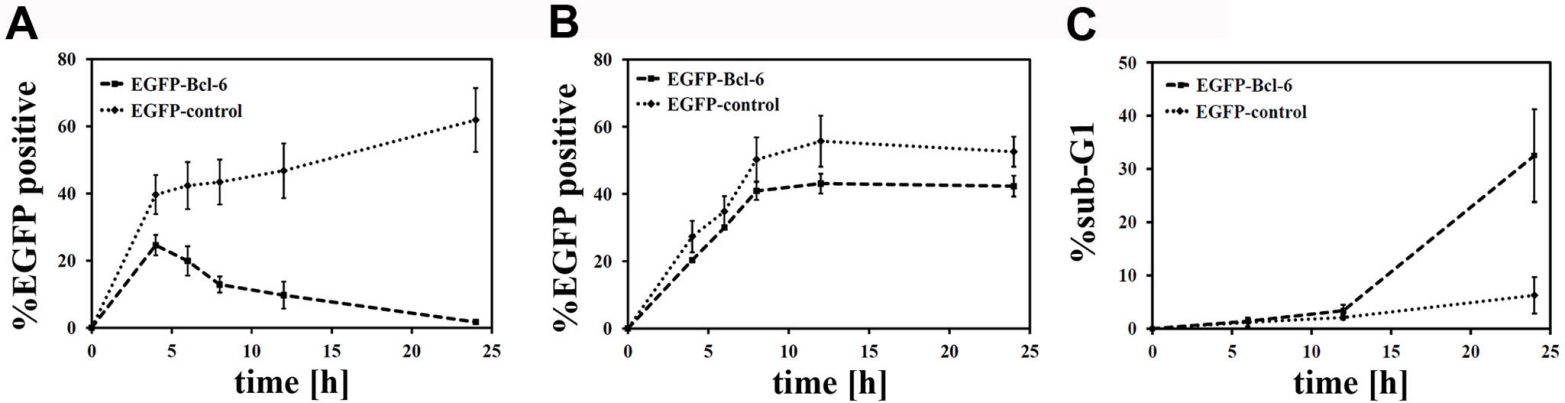
Time course of EGFP-BCL-6 expression in relation to cell death. ECs were transfected with EGFP-BCL-6 or EGFP-C3 control plasmid. Discrimination of dead and live cells was based on the endothelial characteristic that dying cells detach in culture, and detached cells do not survive in suspension. Thus, live (adherent) cells as well as dead (non-adherent) cells were harvested and analyzed by flow cytometry for EGFP fluorescence. The percentage of EGFP-BCL-6 or EGFP positive cells was compared in the live (A) and dead (B) cell populations over a time period of 24 h post transfection. (C) PI staining of the adherent cell population was applied to detect cells with hallmarks of necrosis or late apoptosis (sub G1 DNA content). Only EGFP positive cells were evaluated. Mean values and standard deviations were calculated from two independent experiments.

Regarding the rapid loss of BCL-6/BCoR expression, we further investigated the EGFP-BCL-6 fusion variant (Figure 12A). Comparable to the wildtype BCL-6 molecule, the EGFP-BCL-6 protein showed a transient peak followed by the rapid decrease to baseline within 24 hours. In contrast, the EGFP control protein readily accumulated in transfected ECs over the same time period indicating a negative regulatory mechanism connected to the BCL-6 molecule. To determine whether loss of EGFP-BCL-6 expression was primarily based on downregulation or cytotoxicity, we investigated the dead cell fraction. Transfection of endothelial cells led to a comparable death rate of 22% (+/- 2%) for EGFP-BCL-6 and EGFP gene transfer which was evident by 8 hours and did not further increase. Within the dead cell population about 50% of cells were positive for EGFP or EGFP-BCL-6 (Figure 12B). The observation that there was no accumulation of EGFP-BCL-6 positive cells (when compared to EGFP control) within the dead cell pool, supports the notion that the rapid loss of EGFP-BCL-6 protein seen in endothelial cells is not due to the selective death of cells expressing the EGFP-BCL-6 transgene but rather to a prominent shut-down of gene expression. However, the few cells (2%) remaining positive for EGFP-BCL-6 in culture after 24 h presented with signs of deterioration: one third of cells exhibited loss of cellular DNA, a hallmark of necrosis or late apoptosis (Figure 12C). This indicates that cells which do not downregulate the expression of BCL-6 are prone to cell death. In contrast, EGFP+ control cells at 24 hours showed only a minor fraction of cells with subG1 DNA content.

## Discussion

BCL-6/BCoR are components of a transcriptional repressor complex with reported immunoregulatory functions. Overexpression of either molecule resulted in the formation of nuclear aggregates. Of note, we observed that cells transfected with BCL-6/BCoR expression plasmids exhibited a prominent and rapid downregulation of the respective transcripts as well as proteins which is in line with the notion that nuclear deposition of aggregated proteins may be detrimental [48,49] and hence requires tight control. Thus, the artificial overexpression of these proteins served to reveal this particular protein quality to form nuclear aggregates which may not be observed when BCL-6/BCoR are regulated under physiological conditions. Pathologically elevated levels of BCL-6 have been reported for B-cell lymphoma cells [34]. However, when we analyzed 5 cases of diffuse large B-cell lymphoma (DLBCL) with histologically documented BCL-6 expression, nuclear aggregates were not detectable (Figure S11). This finding may relate to the fact that the expression of BCL-6 observed in vivo does not reach levels achieved by in vitro overexpression. Furthermore, B-cell lymphoma cells are reportedly characterized by high levels of class I as well as class II HDACs [50,51] which may interfere with BCL‑6 aggregate formation as we have observed upon co-expression of various HDAC members.

An in vitro time course experiment showed that nuclear inclusions initiated in the form of punctate aggregates and evolved to form larger, ring-like structures. The inverse relation between diameter and number of rings per cell indicated coalescence of smaller particles into larger spheroids. Life cell imaging to prove merging of aggregates could not be performed because EGFP-fusion of BCL-6 altered the aggregation pattern. However, an inverse relation between size and number of nuclear aggregates has previously been reported for other proteins [31,32] and the preferential fusion of smaller foci to yield larger structures has been documented in this context.

The ring-like appearance of BCL-6/BCoR inclusions in confocal microscopy corresponds to cross-sections of hollow protein spheres. With respect to spherical formations, the interior of aggresomes has been suggested to be filled with associated chaperones or other nuclear components [32]. BCL‑6/BCoR structures were clearly distinct, largely excluding other nuclear components from the inside. Furthermore, we did not detect any association of BCL‑6/BCoR aggregates with molecular chaperones like HSP70 and HSP27 previously reported to be recruited to nuclear inclusions [31,52,53] and BCL‑6/BCoR aggregates were not marked by ubiquitination for proteasomal degradation. Of interest, HSP70 association was triggered upon EGFP-fusion of BCL-6, indicating that the EGFP moiety or altered protein sequence may indeed elicit the cellular stress response. Comparably, overexpression of EGFP (without BCL-6 fusion) resulted in pronounced HSP70 induction and apparent co-localization which seems an important aspect for the frequent application of EGFP-fusion molecules in proteostasis research.

The recruitment of chaperones and components for ubiquitin-mediated proteasomal degradation is generally considered as a defining characteristic of actual “aggresomes”. While we cannot exclude the association of BCL-6/BCoR inclusions with chaperones or proteasomal components not investigated in this study, the lack of association with HSP70 and ubiquitin precludes the term “aggresome” in this context and may in fact reveal a particular feature of BCL-6/BCoR aggregate formation. Mutations and aberrant protein fusions or modifications which trigger changes in protein folding and promote protein aggregation may more readily attract chaperones and proteasomes to eliminate the misfolded proteins. In contrast, the overexpression of BCL-6 and BCoR wild type sequences seems to predominantly induce the assembly in large complexes and may thus not efficiently target the protein to the cellular machinery for protein refolding and degradation.

Overexpression may reportedly increase the likelihood of so-called “domain swapping” thereby promoting protein aggregation. This process has been described for proteins with two independently folded regions separated by a flexible loop which promotes oligomeric interactions [54,55]. BCL-6 presents with a domain structure which may facilitate domain swapping [56], while BCoR constitutes a large molecule [38] with no apparent indication for domain swapping. Of interest, the fact that BCL‑6/BCoR inclusions preferentially develop in the nucleus by a gradual increase in aggregate size, argues against an effect of misfolding or domain swapping during protein synthesis prior to nuclear import. The molecules seem to reach their destined cellular compartment without initial aggregation and deposition in the cytosol, since cytosolic BCL-6/BCoR inclusions are generally rare and arise very late. It has been reported, though, that many proteins require conformational maintenance throughout their “life time” [3] and that the local environment of the cellular compartment may facilitate protein misfolding or oligomerization [15]. Comparably, BCL-6/BCoR aggregation may be controlled or facilitated by nuclear components.

The association with PML bodies is a common hallmark of nuclear aggregates. While it has been suggested to reflect the recruitment of PML complexes to the degradative regions of nuclear inclusions [31,53], it may indeed be involved in the formation of nuclear aggregates. We observed a close association of punctate as well as spheroid BCL-6/BCoR inclusions with PML protein and the rearrangement and accumulation of PML bodies around large aggregates without apparent association of chaperones or proteasomes. Comparably, Fu et al. suggested that the initial protein deposition occurs adjacent to PML bodies, which are subsequently repositioned on the surface of larger nuclear aggregates [31]. With respect to cell cycle distribution, large ring-like structures of BCL-6/BCoR aggregates were restricted to G_0_/G_1_ and S-phase. When exogenous BCL-6 expression was previously investigated in UTA-L cells, a more aggregated BCL‑6 phenotype was similarly reported for S-phase [57,58]. Of particular interest, despite the evident protein overexpression which we observed in dividing cells, BCL-6/BCoR aggregates were not detected during mitosis, a cycle phase where PML bodies are partitioned [59]. This finding strongly argues for the loss or disassembly of aggregate structures during cell division. Furthermore, the appearance of cytosolic inclusions was generally observed in neighboring cells, late in the time course of aggregate formation; and cytosolic inclusions were surrounded by NUPs. We thus propose that BCL‑6/BCoR aggregates are primarily formed in the nucleus but proteins may “exit” to the cytosol upon nuclear envelope breakdown in mitosis and subsequently reassemble in nucleus and cytosol. This mechanism is clearly distinct from the previously suggested mode of aggresome assembly in the cytosol which is believed to occur co-translationally [19] in close contact with HDAC6 and vimentin. In contrast to previously described “nuclear aggresomes” which are found in association with HDAC6 [32] and may thus originate in the cytosol, the nuclear and cytosolic BCL‑6/BCoR aggregates do not co-localize with HDAC6 nor associate with vimentin (data not shown).

Apart from HDAC6, the overexpression of other HDACs did indeed interfere with BCL-6/BCoR aggregate formation: HDACs 1, 4, 5 and 7 which reportedly bind to BCL-6/BCoR [37,38] and which co-localized with BCL-6/BCoR protein in our experiments markedly reduced the appearance of large spheroid structures. Thus, overexpression of these HDACs may interfere with the formation of large BCL‑6/BCoR complexes. Alternatively, BCL-6/BCoR might be subject to protein deacetylation thereby reducing their tendency to aggregate.

To further explore factors of nuclear proteostasis which might prevent or reduce BCL-6/BCoR assembly in large aggregates, we overexpressed members of the HSP family known to control protein folding within the nucleus [6,17]. It has previously been shown that nuclear inclusions may be decreased by the enhanced expression of the molecular chaperone HSP70 [60]. Comparably, we found that co-transfection of BCL-6 and HSP70 expression plasmids resulted in a significantly reduced number of large spheroid structures. The fact that HSP70 co-expression resulted in a reduction rather than a complete abrogation of aggregate formation may be due to limiting endogenous co-factors like HSP40 required for exerting its protective function. The effect of HSP70 overexpression on BCL-6 aggregation was not only based on its chaperone properties but also involved protein degradation by the UPS, as it was partly reversed by proteasome blockade.

In conclusion, our study offers a new perspective on the assembly and remodeling of nuclear aggregates which – distinct from the previously reported nuclear aggresomes - do not involve HDAC6, chaperones, or ubiquitination. These aggregates occur when transcriptional regulators (of wild type sequence) are expressed at high levels, and they may be counterbalanced by co-expression of HDACs or HSP70 to restore nuclear proteostasis.

## Supporting Information

Figure S1 **Nuclear aggregate formation is specific for BCL‑6/BCoR and does not occur upon overexpression of other transcription factors.** ECs were transfected with pCMV4TΔp65 (NF-κB), Sp1 or MeCP2-FLAG expression plasmids. 24 hours later p65 transfected cells were stimulated with 100 ng/ml TNFα for 30 min (to induce nuclear translocation of NF-κB) and were then immunostained with α-p65 antibody. Sp1 and MeCP2-FLAG transfected cells were left untreated and immunostained with α-Sp1 and α-FLAG antibody, respectively. Hoechst 33342 or DRAQ5 were applied to detect nuclear DNA. Scale bars: 5 µm.
(TIF)

Figure S2 **BCoR/BCL-6 aggregate formation is also detected upon protein overexpression in primary fibroblasts or HT-29 colon carcinoma cells.** Cells were transfected with EFp-BCL-6 and/or EFp-BCoR-A expression plasmid and immunostained with α-BCL-6 and α-BCoR antibodies after 24 hours (fibroblasts) or 48 hours (HT-29). (A) Separate expression of BCL-6 and BCoR was compared to concomitant overexpression (B). Protein co-localization is indicated in yellow. Nuclear DNA is detected by Hoechst 33342 stain. Scale bars: 5 µm.
(TIF)

Figure S3 **BCL-6/BCoR aggregates are detected independent of immunocytochemical preparation method.** 24 hours after transfection with EFp-BCL-6 or EFp-BCoR-A expression plasmids ECs were fixed and permeabilized using different agents: 3.7% formaldehyde and 0.5% Triton X-100; 4% paraformaldehyde and 0.5% Triton X-100; 4% paraformaldehyde and ice-cold methanol; ice-cold methanol and acetone; PEM-buffer and ice-cold ethanol. BCL-6 and BCoR were visualized with α-BCL-6 and α-BCoR antibodies, respectively. DRAQ5 was applied to detect nuclear DNA. Although the use of alcohols, in particular methanol in combination with acetone led to loss of nuclear matrix signal, BCL-6/BCoR aggregates were readily detectable. Formaldehyde or paraformaldehyde fixation and cell permeabilization by Triton X-100 proved to be optimal for detection of BCL‑6/BCoR protein. Scale bars: 5 µm.
(TIF)

Figure S4 **BCL-6/BCoR aggregates do not associate with or alter nucleolar structures.** ECs were transfected with EFp-BCL-6 or EFp-BCoR-A plasmid and immunostained with α-BCL-6 or α-BCoR antibody in combination with α-nucleolin (C23) antibody at 12 hours after transfection. Nuclei were visualized by DNA stain Hoechst 33342. Scale bar: 5 µm (applying to all panels).
(TIF)

Figure S5 **BCL-6/BCoR aggregates do not induce or co-localize with HSP27.** ECs were transfected with EFp-BCL-6, EFp-BCoR-A or EGFP-BCL-6 plasmid and immunostained with α-BCL-6 or α-BCoR antibody in combination with α-HSP27 antibody at 12 hours after transfection. Nuclei were visualized by DNA stain Hoechst 33342. Regions of protein co-localization are indicated in yellow. Scale bar: 5 µm (applying to all panels).
(TIF)

Figure S6 **BCL-6/BCoR aggregates are not marked by ubiquitin.** EC transfection with EFp-BCL-6, EFp-BCoR-A or EGFP-BCL-6 and culture for 12 h was followed by processing with Hoechst 33342 and antibodies against BCL-6, BCoR and ubiquitin for CLSM imaging. Arrows point to the accumulation of ubiquitin around perinuclear EGFP-BCL-6 aggregates. Scale bar: 5 µm (applying to all panels).
(TIF)

Figure S7 **BCL-6/BCoR aggregates do not co-localize with endogenous PPARδ or Sp1.** ECs were transfected with EFp-BCL-6 or EFp-BCoR-A plasmid and processed for CLSM imaging after 24 hours. (A) Co-staining of BCoR or BCL-6 with PPARδ antibodies. (B) Co-staining of BCL-6 or BCoR with Sp1 antibodies. Nuclei were visualized by DNA stain Hoechst 33342. Representative cells with small punctate or large ring-like aggregates were chosen for BCL-6 and BCoR overexpression. Control images show endogenous Sp1 and PPARδ in non-transfected ECs. Scale bars: 5 µm.
(TIF)

Figure S8 **Transcriptional repression in the presence of BCL-6 aggregates.** ECs were co-transfected with a luciferase reporter construct carrying five BCL-6 binding sites and with the EFp-BCL-6 expression plasmid or EFp-Link control vector. Firefly luciferase activity was measured at 6, 12 and 24 hours after transfection and is expressed in relation to EFp-Link control samples set to 100%. Data shown represent the mean and standard deviation of three independent experiments. *, p < 0.05; **, p < 0.01; ***, p < 0.001 (T-test).
(TIF)

Figure S9 **Association of BCL-6/BCoR aggregates with nuclear Cajal bodies.** ECs were transfected with EFp-BCL-6 or EFp-BCoR-A plasmid and cultured for 24 h. For immunocytochemistry, antibodies against BCL-6 or BCoR were applied in combination with α-coilin antibody. Associations of the coilin protein (representing Cajal bodies) with large BCL-6 inclusions are marked by arrows. Arrowheads indicate co-localization of Cajal bodies with BCoR aggregates. Scale bars: 5 µm.
(TIF)

Figure S10 **Nuclear lamin B1 distribution is differentially affected by BCL-6 and BCoR aggregates.** CLSM images were acquired of ECs 24 hours after transfection with EFp-BCL-6 or EFp-BCoR-A plasmid and immunostaining with antibodies against lamin B1, BCL-6 or BCoR. DRAQ5 was applied to detect nuclear DNA. Regions of co-localization are shown in yellow. Scale bars: 5 µm.
(TIF)

Figure S11 **DLBCL cells express high levels of BCL-6 without nuclear aggregate formation.** Five cases of diffuse large B-cell lymphoma with histologically documented BCL-6 expression were chosen for CLSM analysis. Tissue sections were stained with α-BCL-6 (red) and α-CD45 antibody (green) to mark leukocyte membranes. Nuclei were visualized by DNA stain Hoechst 33342. A representative tissue section is shown. Careful screening of all samples revealed high BCL-6 expression in lymphoma cells but did not detect nuclear BCL-6 aggregates. Scale bar: 50 µm.
(TIF)
