# Supplementary figures and images for: Overexpression of the Transcriptional Repressor Complex BCL-6/BCoR Leads to Nuclear Aggregates Distinct from Classical Aggresomes
Source: PLoS One. 2013 Oct 11;8(10):e76845. doi: 10.1371/journal.pone.0076845 (PMC3795655; doi:10.1371/journal.pone.0076845)

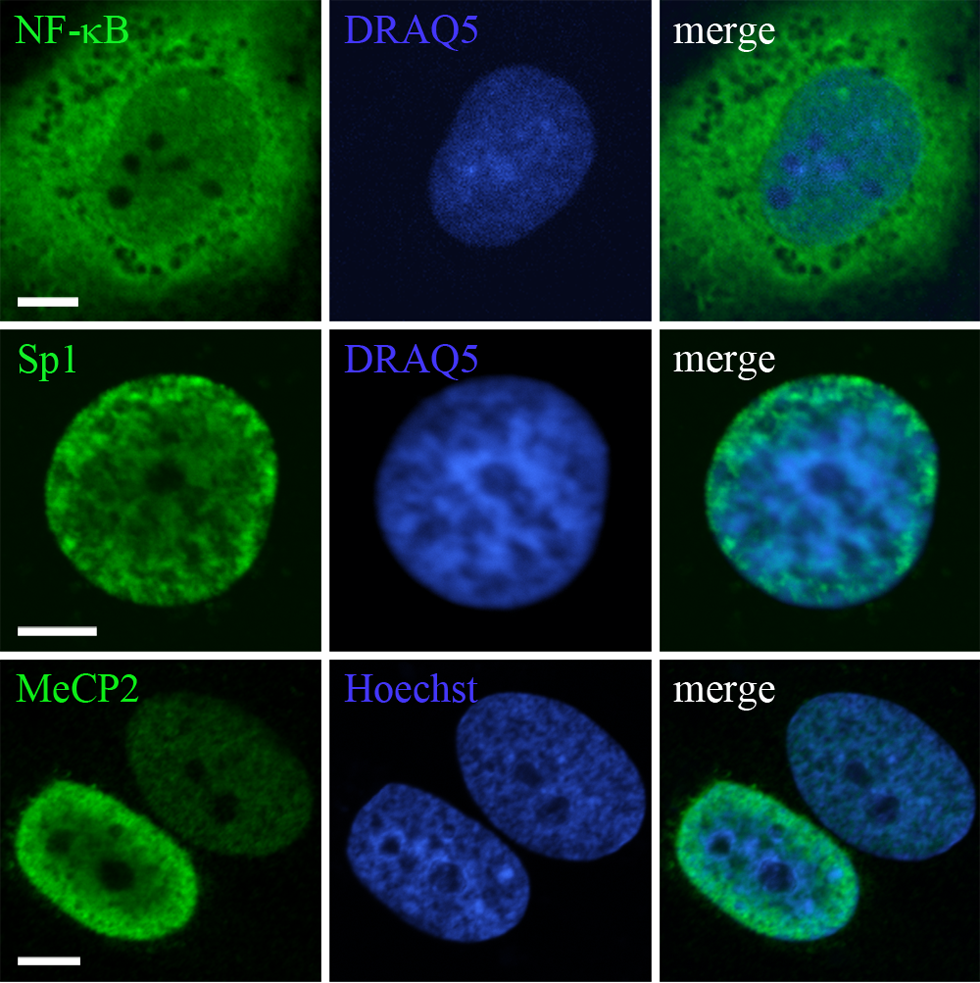

Supplement: Figure S1 — Nuclear aggregate formation is specific for BCL‑6/BCoR and does not occur upon overexpression of other transcription factors. ECs were transfected with pCMV4TΔp65 (NF-κB), Sp1 or MeCP2-FLAG expression plasmids. 24 hours later p65 transfected cells were stimulated with 100 ng/ml TNFα for 30 min (to induce nuclear translocation of NF-κB) and were then immunostained with α-p65 antibody. Sp1 and MeCP2-FLAG transfected cells were left untreated and immunostained with α-Sp1 and α-FLAG antibody, respectively. Hoechst 33342 or DRAQ5 were applied to detect nuclear DNA. Scale bars: 5 µm. (TIF) [file pone.0076845.s001.tif]

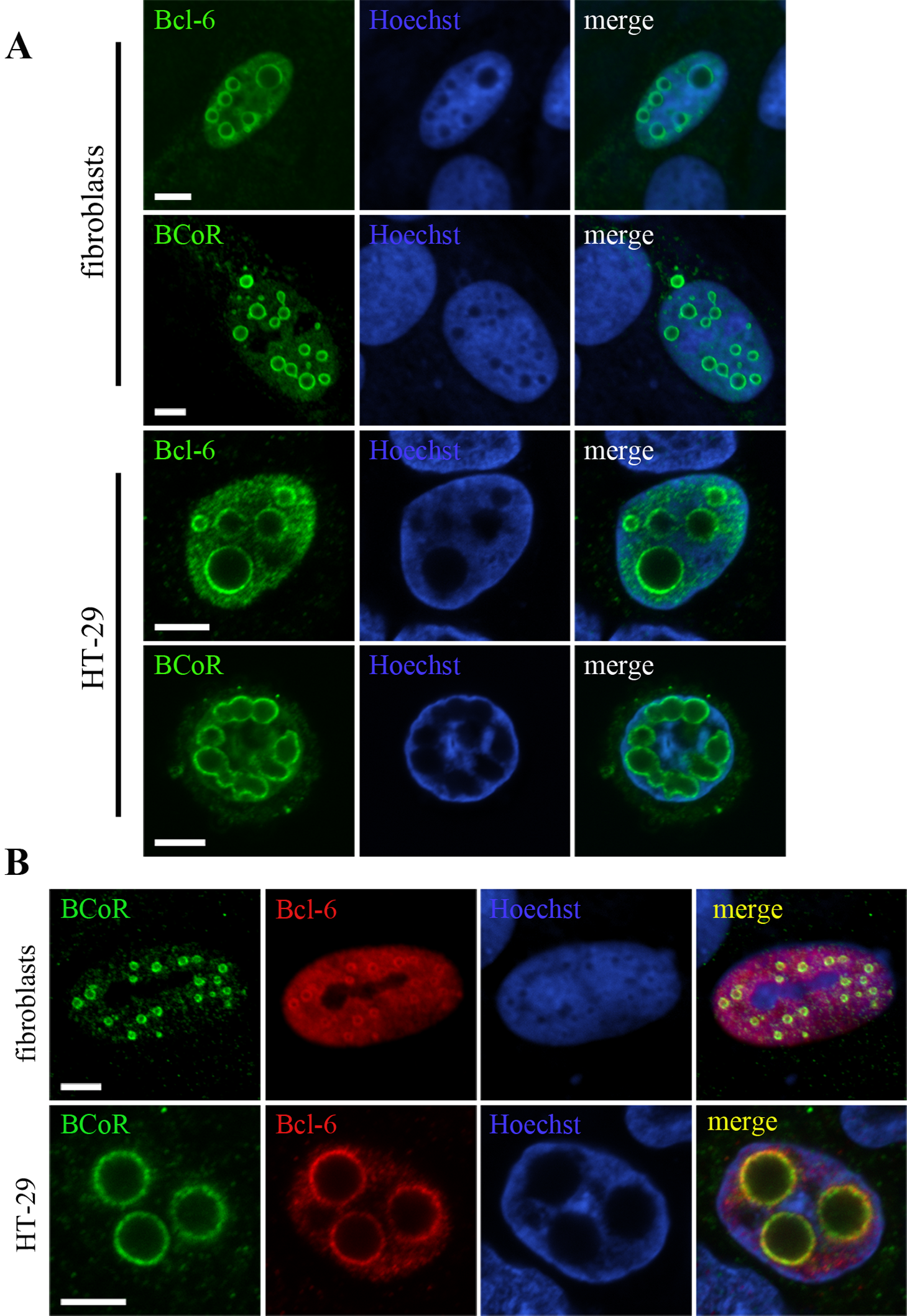

Supplement: Figure S2 — BCoR/BCL-6 aggregate formation is also detected upon protein overexpression in primary fibroblasts or HT-29 colon carcinoma cells. Cells were transfected with EFp-BCL-6 and/or EFp-BCoR-A expression plasmid and immunostained with α-BCL-6 and α-BCoR antibodies after 24 hours (fibroblasts) or 48 hours (HT-29). (A) Separate expression of BCL-6 and BCoR was compared to concomitant overexpression (B). Protein co-localization is indicated in yellow. Nuclear DNA is detected by Hoechst 33342 stain. Scale bars: 5 µm. (TIF) [file pone.0076845.s002.tif]

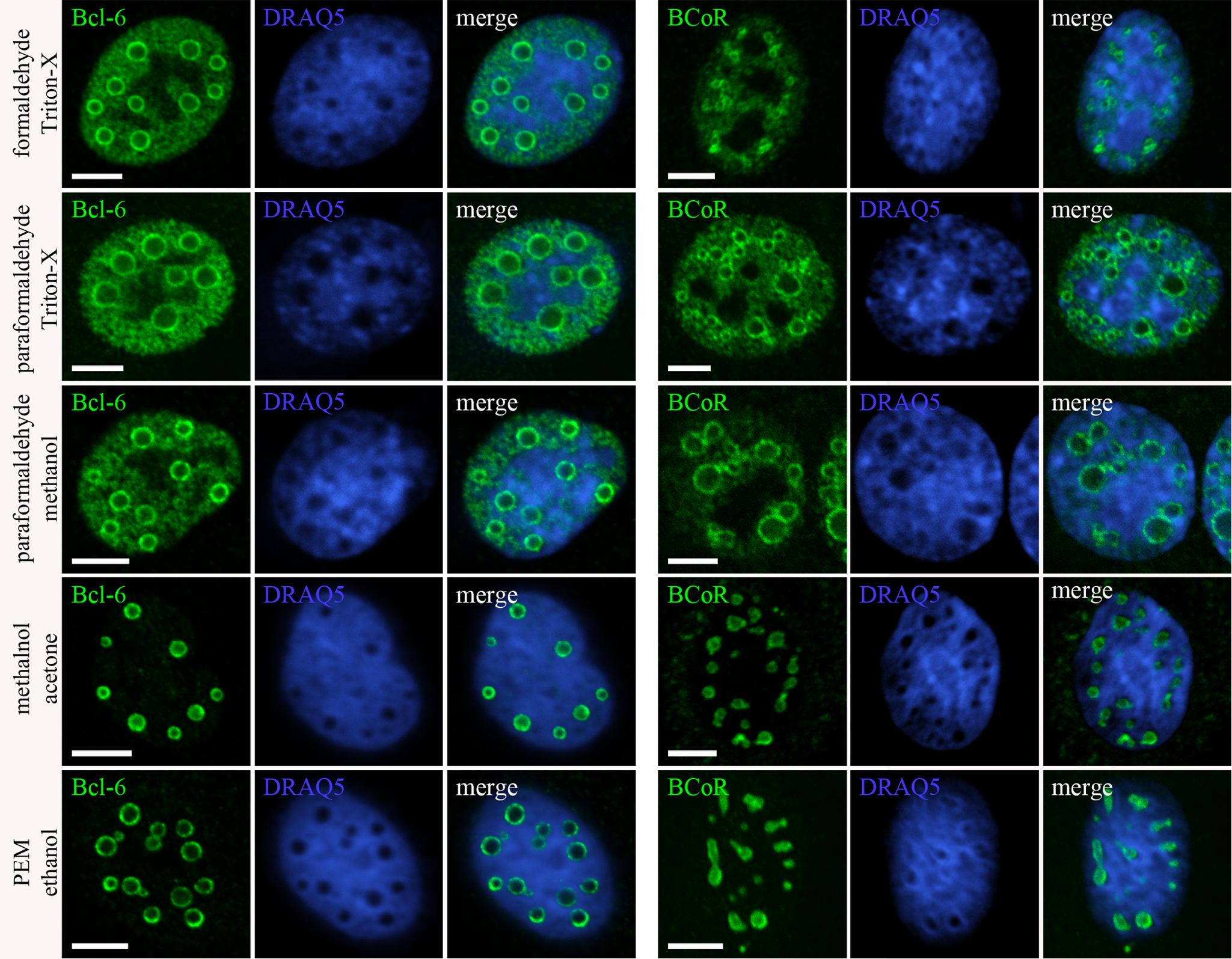

Supplement: Figure S3 — BCL-6/BCoR aggregates are detected independent of immunocytochemical preparation method. 24 hours after transfection with EFp-BCL-6 or EFp-BCoR-A expression plasmids ECs were fixed and permeabilized using different agents: 3.7% formaldehyde and 0.5% Triton X-100; 4% paraformaldehyde and 0.5% Triton X-100; 4% paraformaldehyde and ice-cold methanol; ice-cold methanol and acetone; PEM-buffer and ice-cold ethanol. BCL-6 and BCoR were visualized with α-BCL-6 and α-BCoR antibodies, respectively. DRAQ5 was applied to detect nuclear DNA. Although the use of alcohols, in particular methanol in combination with acetone led to loss of nuclear matrix signal, BCL-6/BCoR aggregates were readily detectable. Formaldehyde or paraformaldehyde fixation and cell permeabilization by Triton X-100 proved to be optimal for detection of BCL‑6/BCoR protein. Scale bars: 5 µm. (TIF) [file pone.0076845.s003.tif]

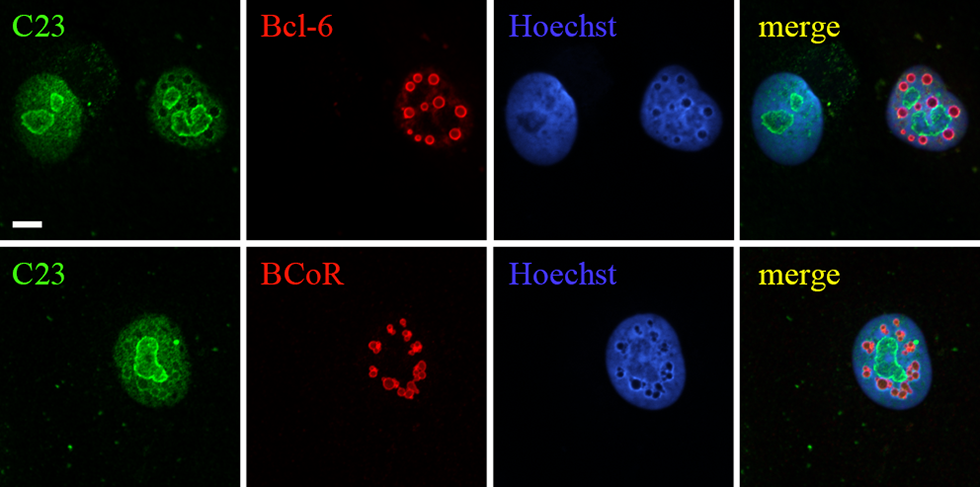

Supplement: Figure S4 — BCL-6/BCoR aggregates do not associate with or alter nucleolar structures. ECs were transfected with EFp-BCL-6 or EFp-BCoR-A plasmid and immunostained with α-BCL-6 or α-BCoR antibody in combination with α-nucleolin (C23) antibody at 12 hours after transfection. Nuclei were visualized by DNA stain Hoechst 33342. Scale bar: 5 µm (applying to all panels). (TIF) [file pone.0076845.s004.tif]

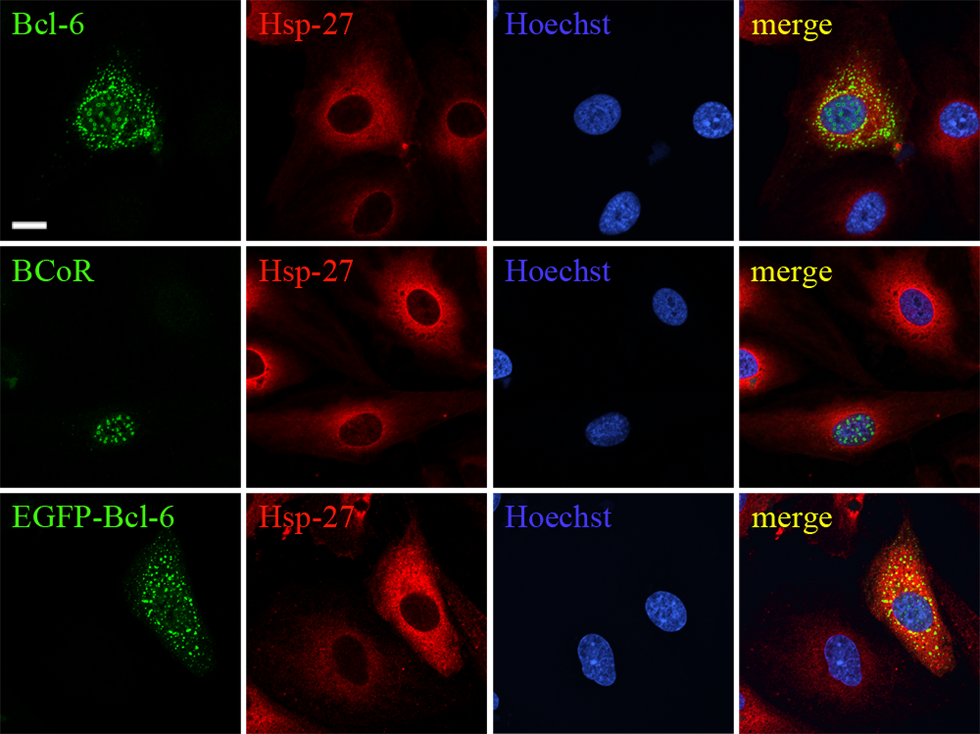

Supplement: Figure S5 — BCL-6/BCoR aggregates do not induce or co-localize with HSP27. ECs were transfected with EFp-BCL-6, EFp-BCoR-A or EGFP-BCL-6 plasmid and immunostained with α-BCL-6 or α-BCoR antibody in combination with α-HSP27 antibody at 12 hours after transfection. Nuclei were visualized by DNA stain Hoechst 33342. Regions of protein co-localization are indicated in yellow. Scale bar: 5 µm (applying to all panels). (TIF) [file pone.0076845.s005.tif]

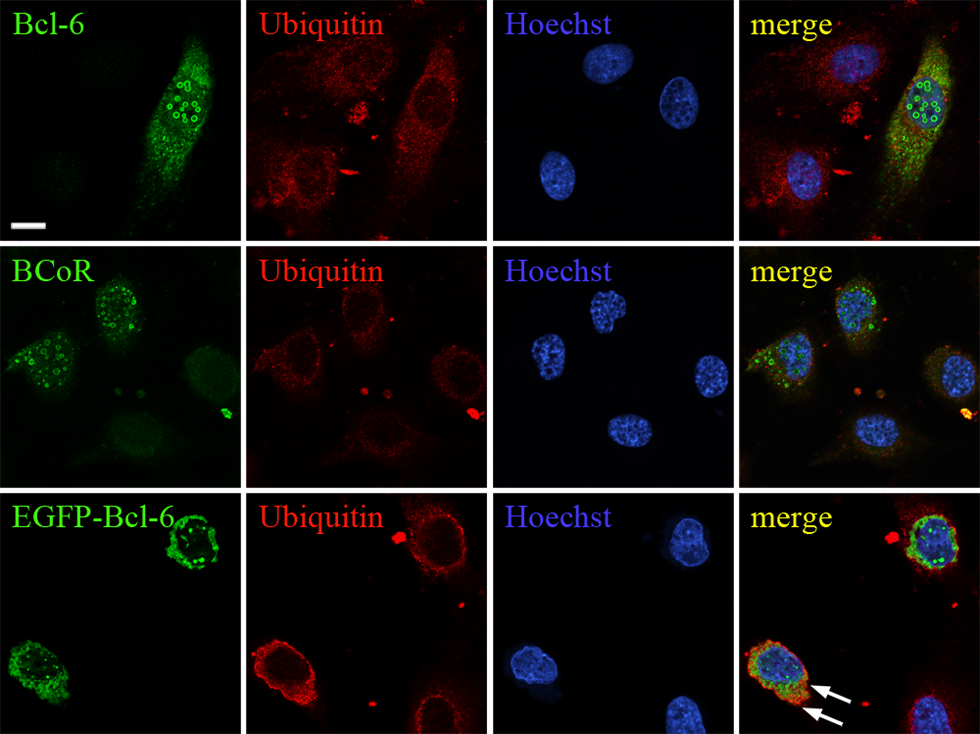

Supplement: Figure S6 — BCL-6/BCoR aggregates are not marked by ubiquitin. EC transfection with EFp-BCL-6, EFp-BCoR-A or EGFP-BCL-6 and culture for 12 h was followed by processing with Hoechst 33342 and antibodies against BCL-6, BCoR and ubiquitin for CLSM imaging. Arrows point to the accumulation of ubiquitin around perinuclear EGFP-BCL-6 aggregates. Scale bar: 5 µm (applying to all panels). (TIF) [file pone.0076845.s006.tif]

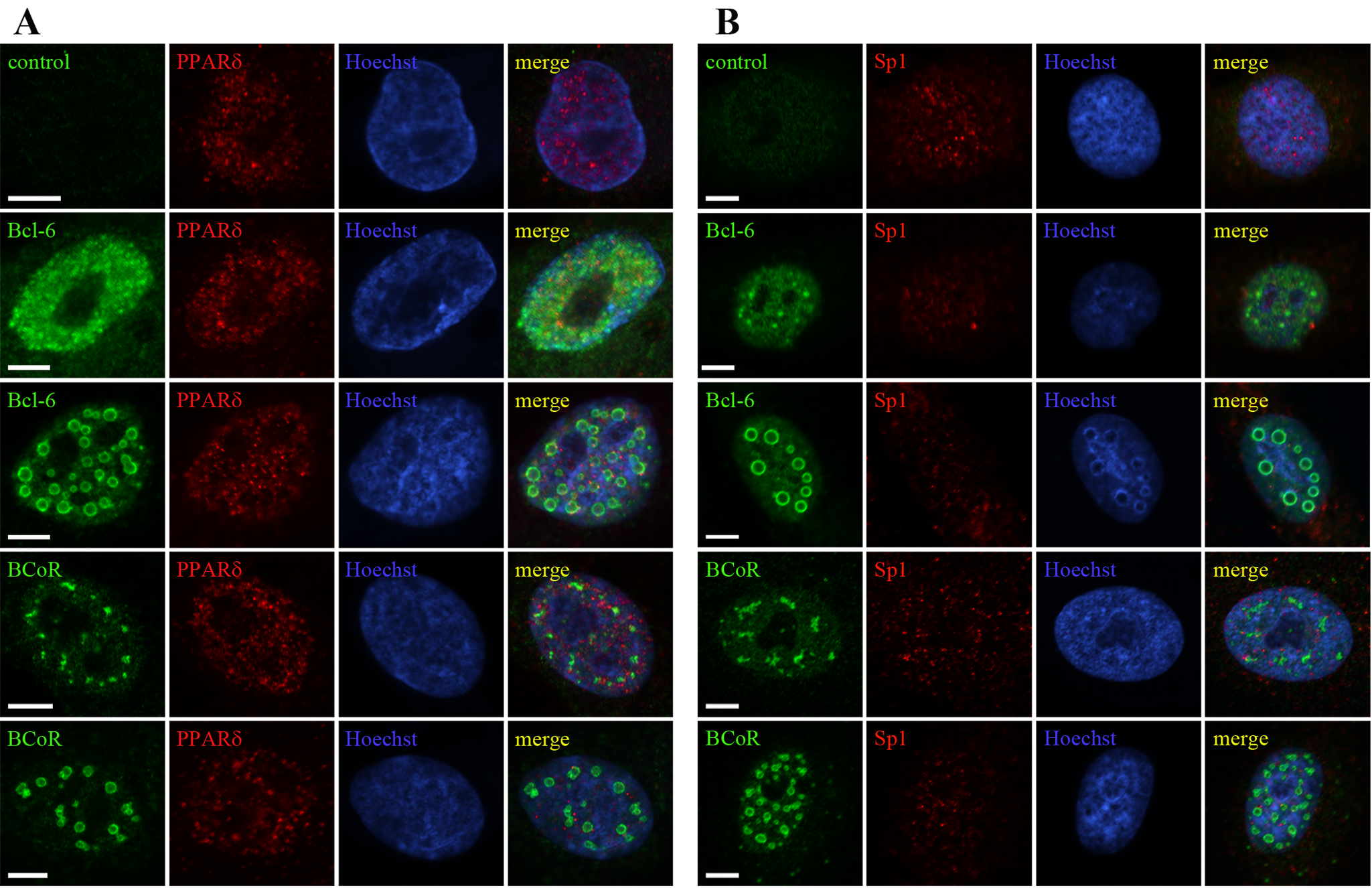

Supplement: Figure S7 — BCL-6/BCoR aggregates do not co-localize with endogenous PPARδ or Sp1. ECs were transfected with EFp-BCL-6 or EFp-BCoR-A plasmid and processed for CLSM imaging after 24 hours. (A) Co-staining of BCoR or BCL-6 with PPARδ antibodies. (B) Co-staining of BCL-6 or BCoR with Sp1 antibodies. Nuclei were visualized by DNA stain Hoechst 33342. Representative cells with small punctate or large ring-like aggregates were chosen for BCL-6 and BCoR overexpression. Control images show endogenous Sp1 and PPARδ in non-transfected ECs. Scale bars: 5 µm. (TIF) [file pone.0076845.s007.tif]

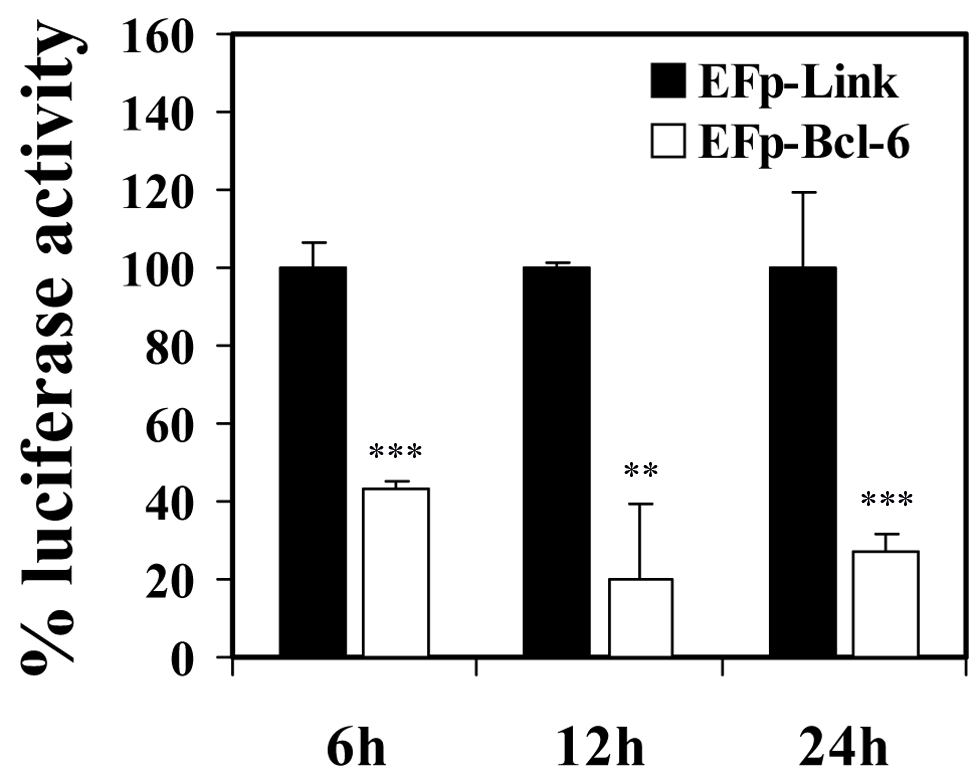

Supplement: Figure S8 — Transcriptional repression in the presence of BCL-6 aggregates. ECs were co-transfected with a luciferase reporter construct carrying five BCL-6 binding sites and with the EFp-BCL-6 expression plasmid or EFp-Link control vector. Firefly luciferase activity was measured at 6, 12 and 24 hours after transfection and is expressed in relation to EFp-Link control samples set to 100%. Data shown represent the mean and standard deviation of three independent experiments. *, p < 0.05; **, p < 0.01; ***, p < 0.001 (T-test). (TIF) [file pone.0076845.s008.tif]

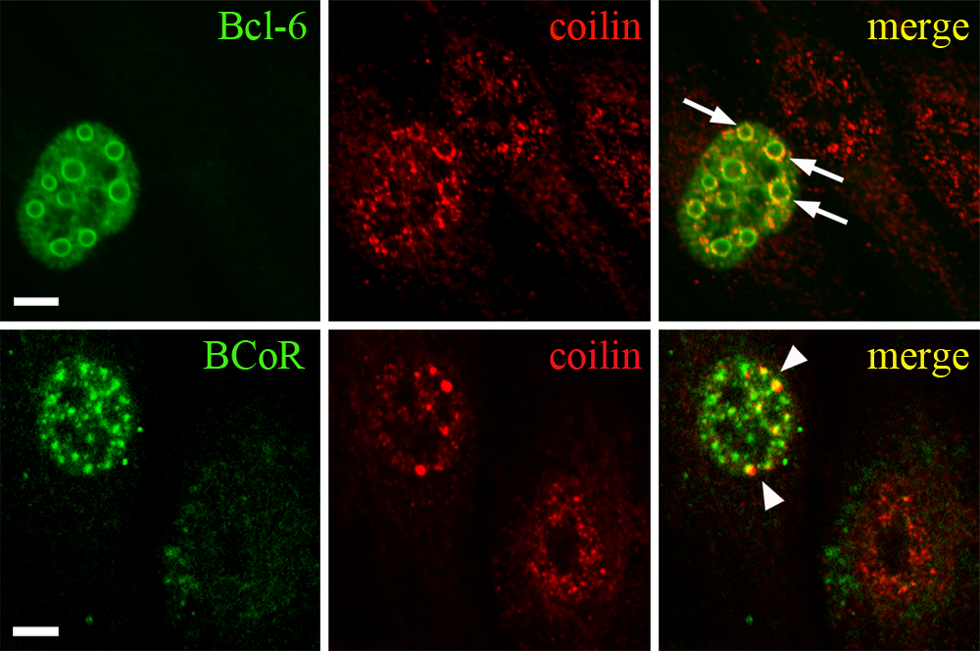

Supplement: Figure S9 — Association of BCL-6/BCoR aggregates with nuclear Cajal bodies. ECs were transfected with EFp-BCL-6 or EFp-BCoR-A plasmid and cultured for 24 h. For immunocytochemistry, antibodies against BCL-6 or BCoR were applied in combination with α-coilin antibody. Associations of the coilin protein (representing Cajal bodies) with large BCL-6 inclusions are marked by arrows. Arrowheads indicate co-localization of Cajal bodies with BCoR aggregates. Scale bars: 5 µm. (TIF) [file pone.0076845.s009.tif]

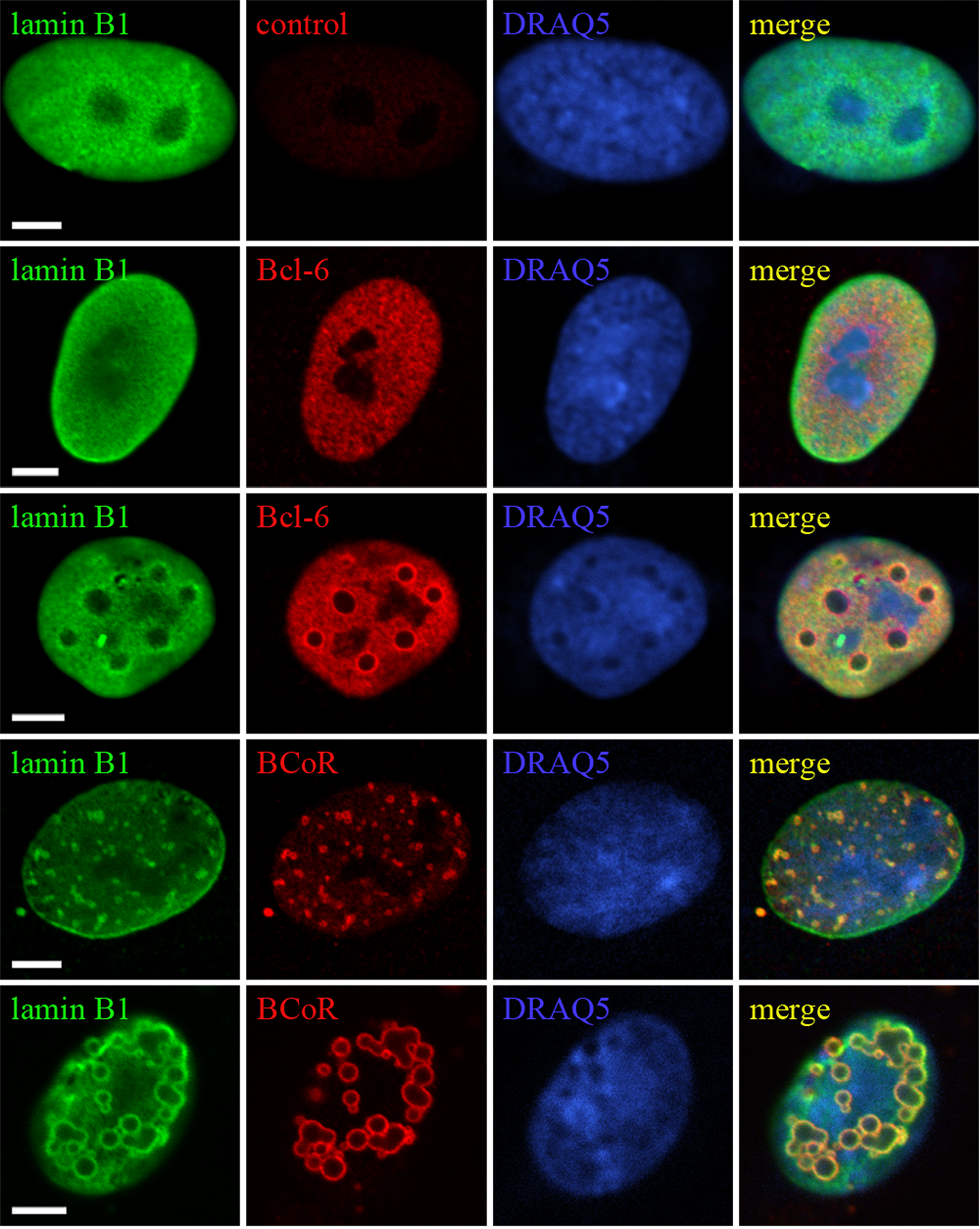

Supplement: Figure S10 — Nuclear lamin B1 distribution is differentially affected by BCL-6 and BCoR aggregates. CLSM images were acquired of ECs 24 hours after transfection with EFp-BCL-6 or EFp-BCoR-A plasmid and immunostaining with antibodies against lamin B1, BCL-6 or BCoR. DRAQ5 was applied to detect nuclear DNA. Regions of co-localization are shown in yellow. Scale bars: 5 µm. (TIF) [file pone.0076845.s010.tif]

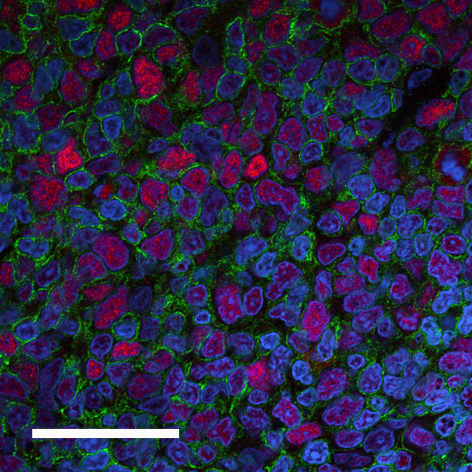

Supplement: Figure S11 — DLBCL cells express high levels of BCL-6 without nuclear aggregate formation. Five cases of diffuse large B-cell lymphoma with histologically documented BCL-6 expression were chosen for CLSM analysis. Tissue sections were stained with α-BCL-6 (red) and α-CD45 antibody (green) to mark leukocyte membranes. Nuclei were visualized by DNA stain Hoechst 33342. A representative tissue section is shown. Careful screening of all samples revealed high BCL-6 expression in lymphoma cells but did not detect nuclear BCL-6 aggregates. Scale bar: 50 µm. (TIF) [file pone.0076845.s011.tif]
